# Rituximab versus cyclophosphamide for the treatment of connective tissue disease-associated interstitial lung disease (RECITAL): study protocol for a randomised controlled trial

**DOI:** 10.1186/s13063-017-2016-2

**Published:** 2017-06-15

**Authors:** Peter Saunders, Vicky Tsipouri, Gregory J. Keir, Deborah Ashby, Marcus D. Flather, Helen Parfrey, Daphne Babalis, Elisabetta A. Renzoni, Christopher P. Denton, Athol U. Wells, Toby M. Maher

**Affiliations:** 1 NIHR Biomedical Research Unit, Royal Brompton Hospital, Sydney Street, London, SW3 6NP UK; 2 Princess Alexandra Hospital, Brisbane, QLD Australia; 3 Imperial Clinical Trials Unit, School of Public Health, Imperial College London, St. Mary’s Campus, Norfolk Place, London, W2 1PG UK; 4 Norwich Medical School, University of East Anglia, Norfolk and Norwich University Hospital, Norwich, UK; 5 Respiratory Medicine Division, Department of Medicine, University of Cambridge School of Clinical Medicine, Addenbrooke’s Hospital, CUHNHSFT and Papworth Hospital NHS Foundation Trust, Cambridge, UK; 6 Fibrosis Research Group, National Heart and Lung Institute, Imperial College London, Sir Alexander Fleming Building, Imperial College, London, SW7 2AZ UK; 7 Centre for Rheumatology and Connective Tissue Diseases, Royal Free Campus, University College London, London, NW3 2PF UK

**Keywords:** Myositis, Scleroderma, Mixed connective tissue disease, Pulmonary fibrosis, Respiratory failure, Biomarkers

## Abstract

**Background:**

Interstitial lung disease (ILD) frequently complicates systemic autoimmune disorders resulting in considerable morbidity and mortality. The connective tissue diseases (CTDs) most frequently resulting in ILD include: systemic sclerosis, idiopathic inflammatory myositis (including dermatomyositis, polymyositis and anti-synthetase syndrome) and mixed connective tissue disease. Despite the development, over the last two decades, of a range of biological therapies which have resulted in significant improvements in the treatment of the systemic manifestations of CTD, the management of CTD-associated ILD has changed little. At present there are no approved therapies for CTD-ILD. Following trials in scleroderma-ILD, cyclophosphamide is the accepted standard of care for individuals with severe or progressive CTD-related ILD. Observational studies have suggested that the anti-CD20 monoclonal antibody, rituximab, is an effective rescue therapy in the treatment of refractory CTD-ILD. However, before now, there have been no randomised controlled trials assessing the efficacy of rituximab in this treatment population.

**Methods/design:**

RECITAL is a UK, multicentre, prospective, randomised, double-blind, double-dummy, controlled trial funded by the Efficacy and Mechanism Evaluation Programme of the Medical Research Council and National Institute for Health Research. The trial will compare rituximab 1 g given intravenously, twice at an interval of 2 weeks, with intravenously administered cyclophosphamide given monthly at a dose of 600 mg/m^2^ body surface area in individuals with ILD due to systemic sclerosis, idiopathic inflammatory myositis (including anti-synthetase syndrome) or mixed connective tissue disease. A total of 116 individuals will be randomised 1:1 to each of the two treatment arms, with stratification based on underlying CTD, and will be followed for a total of 48 weeks from first dose. The primary endpoint for the study will be change in forced vital capacity (FVC) at 24 weeks. Key secondary endpoints include: safety, change in FVC at 48 weeks as well as survival, change in oxygen requirements, total 48-week corticosteroid exposure and utilisation of health care resources.

**Discussion:**

This is the first randomised control trial to study the efficacy of rituximab as first-line treatment in CTD-associated ILD. The results generated should provide important information on the treatment of a life-threatening complication affecting a rare group of CTDs.

**Trial registration:**

ClinicalTrials.gov, NCT01862926. Registered on 22 May 2013.

**Electronic supplementary material:**

The online version of this article (doi:10.1186/s13063-017-2016-2) contains supplementary material, which is available to authorized users.

## Background

Interstitial lung disease (ILD) is characterised by inflammation and/or fibrosis that results in thickening and distortion of the alveolar wall with consequent impairment of gas exchange. Affected individuals typically present with progressive breathlessness which frequently causes respiratory failure and death. There are many described causes of ILD; however, one of the commonest is that resulting from lung involvement by systemic autoimmune diseases [1]. This group of conditions, the connective tissue diseases (CTDs), is an important cause of disability and death in the working-age population. Over the last decade, improvements in therapy for the CTDs have seen the prognosis for individuals with these conditions dramatically improve. Despite these improvements in care there has been little, if any, change in therapy for ILD occurring as a consequence of CTD, with respiratory disease growing in importance in these patients. For many CTD sufferers, disease-associated ILD is now the major cause of disability and exercise limitation, whilst in systemic sclerosis it is now the principal cause of mortality [2].

The pathogenesis of CTD-ILD is complex and poorly understood. It is, however, generally accepted that underlying immune system dysfunction and immune-mediated pulmonary inflammation are critical to CTD-ILD development and progression. Abnormalities of cellular and humoral immune function have been described in ILD associated with systemic sclerosis (SSc) [3–5], idiopathic inflammatory myopathy and several other CTDs. The mechanisms leading on to fibrosis remain poorly understood, as do the factors that determine which individuals with CTD develop ILD. Nonetheless, evidence from treatment trials suggests that the modulation of inflammation with immunosuppressant therapies, particularly cyclophosphamide, results in some regression of ILD and prevents the development of further fibrosis.

Different CTDs manifest varying forms of ILD. Individuals with scleroderma and mixed connective tissue disease (MCTD) most commonly develop the histological lesion of nonspecific interstitial pneumonia (NSIP). Those with idiopathic inflammatory myositis typically have combined organising pneumonia and nonspecific interstitial pneumonia (NSIP). By contrast, individuals with rheumatoid disease frequently have fibrosis with the histological pattern of usual interstitial pneumonia (UIP) and tend to be resistant to therapy with high-dose immunosuppression.

Currently, standard of care for severe, progressive CTD-ILD includes immunosuppression with intravenous cyclophosphamide (600 mg/m^2^) administered monthly for 6 months, followed by maintenance oral immunosuppression [6, 7]. Occasionally, this intensive immunosuppressive therapy fails to control pulmonary inflammation and alternative therapies may be required. Rituximab, a chimeric (human/mouse) monoclonal antibody with a high affinity for the CD20 surface antigen expressed on pre-B and B-lymphocytes, results in rapid depletion of B-cells from the peripheral circulation for 6 to 9 months [8, 9]. Evidence for the effectiveness of B-cell depletion exists in a number of immune-mediated conditions, including rheumatoid arthritis [10–12], ANCA-associated vasculitis [13, 14] and immune thrombocytopenic purpura [15]. Several case series suggest that rituximab may also be effective in ILD occurring in the context of immunological overactivity, with favourable responses reported in anti-synthetase-associated ILD [16] and scleroderma-ILD [17, 18]. Our own experience has demonstrated rituximab to be an effective, potentially life-saving therapeutic intervention in the treatment of very severe, progressive CTD-ILD unresponsive to conventional immunosuppression [19, 20].

It is hoped that this study will advance the standard of care for those with CTD-ILD. Despite current best treatment, individuals with extensive ILD due to scleroderma have a median survival of less than 5 years and a similar poor prognosis is observed in individuals with inflammatory myositis and MCTD-associated ILD [21]. If rituximab can be shown to improve 6-month and 1-year lung function in this group then it is to be hoped that this will translate in to improvements in longer-term survival and associated reductions in morbidity. The simplified dosing regimen for rituximab when compared to cyclophosphamide also affords the potential for reducing the burden on patients (and their carers) of frequent hospital attendances. Similarly, although drug costs are higher for rituximab, it is hoped that a full economic costing will demonstrate savings based on reduced utilisation of health care resources and fewer hospital visits. There are currently no available biomarkers for assessing response to therapy or risk of disease progression in CTD-ILD. By closely studying patients in each treatment arm and undertaking exploratory biomarker analysis it is hoped that we might identify potential disease- and therapy-specific biomarkers for future development and use in clinical practice.

Against the potential benefits must be balanced the risks of treatment. As noted, rituximab is a well-established therapy for a range of indications and, as such, its safety profile is well known. Potential risks of therapy include; infusion reactions, infection, arthralgia and hypercholesterolaemia. Very rarely, long-term hypogammaglobulinaemia and neutropaenia have been reported. These side effects can be balanced against those known to occur following cyclophosphamide which include haemorrhagic cystitis, nausea and vomiting and ,in the longer term, an increased incidence of bladder malignancy.

### Aims and objectives

The overall aims of this study are to:

1. Demonstrate that intravenously administered rituximab has superior efficacy to current best treatment (intravenous cyclophosphamide) for CTD-ILD
2. Compare the safety profile of rituximab to intravenously administered cyclophosphamide in individuals with CTD-ILD
3. Assess the health economic benefits of rituximab compared to current standard of care for CTD-ILD, and
4. Evaluate a range of exploratory biomarkers for disease severity, prognosis and treatment response in CTD-ILD

## Methods/design

**Fig. 1:**
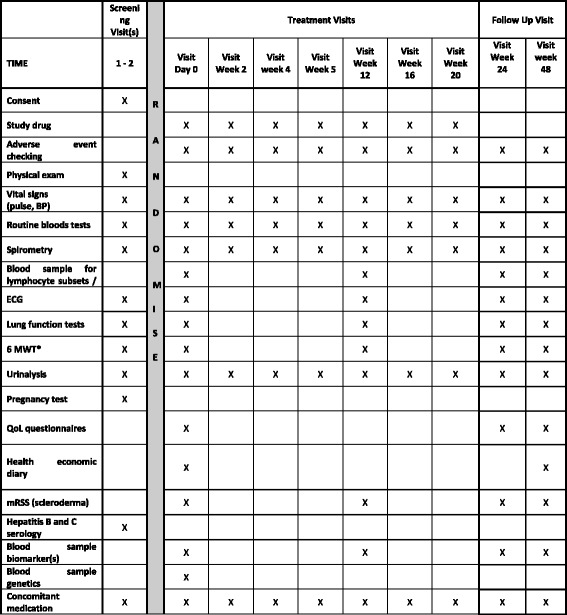
Standard Protocol Items: Recommendations for Interventional Trials (SPIRIT) figure. *BP* blood pressure, *ECG* electrocardiogram, *6MWT* 6-min walk test, *QoL* quality of life, *mRSS* modified Rodnan Skin Score

The RECITAL study is a UK, multicentre, prospective, randomised, double-blind, double-dummy trial of intravenously administered rituximab compared with intravenously administered cyclophosphamide in patients with severe, progressive CTD-ILD. Patients will be randomised to two groups. Both groups will receive placebo to match the different regimens. The study design is outlined in Table 1 and Fig. 1. More details are available in the RECITAL protocol, version 6.1, dated 15 December 2014.

**Table 1:** Outline of planned treatment interventions by visit

	Rituximab group	Cyclophosphamide group
Day 0	IV active rituximab 1000 mg	IV 600 mg mg/m^2^ body surface area
Day 14	IV active rituximab 1000 mg	Placebo
Week 4	Placebo	IV 600 mg mg/m^2^ body surface area
Week 8	Placebo	IV 600 mg mg/m^2^ body surface area
Week 12	Placebo	IV 600 mg mg/m^2^ body surface area
Week 16	Placebo	IV 600 mg mg/m^2^ body surface area
Week 20	Placebo	IV 600 mg mg/m^2^ body surface area

*IV* intravenously administered

### Location and setting

RECITAL is sponsored by the Royal Brompton and Harefield NHS Foundation Trust and will recruit subjects from eight to twelve UK centres all with expertise in both ILD and rheumatological disorders.

### Study population and eligibility criteria

A total of 116 subjects will be enrolled. Subjects should fulfil the following criteria:

- A diagnosis of connective tissue disease (CTD), based on internationally accepted criteria, in one of the following categories [22–25]:
  - ○ Systemic sclerosis
  - ○ Idiopathic interstitial myopathy (including polymyositis/dermatomyositis)
  - ○ Mixed connective tissue disease (MCTD)
- Severe and/or progressive interstitial lung disease (ILD) associated with the underlying CTD
- Chest high-resolution computed tomography (HRCT) performed within 12 months of randomisation
- Intention of the caring physician to treat the ILD with intravenously administered cyclophosphamide (with treatment indications including: deteriorating symptoms attributable to ILD, deteriorating lung function tests, worsening gas exchange or extent of ILD at first presentation) and where there is a reasonable expectation that immunosuppressive treatment will stabilise or improve CTD-ILD. In individuals with scleroderma it is anticipated that patients will fulfil the criteria for extensive disease defined by Goh et al. [21]
- Able to provide written informed consent

Subjects should not enter the study if any of the exclusion criteria listed in Additional file 1 are fulfilled.

### Interventions

Cyclophosphamide will be administered by intravenous infusion at a dose of 600 mg/m^2^ body surface area (BSA). The dose will be repeated every 4 weeks for a total of six doses. If clinically required, individual doses may be delayed by up to 10 days. If longer delay is required the planned dose should be omitted and the next scheduled dose given. Body surface area will be calculated with baseline measurements using the Mosteller method with dose modification for any subjects with a Body Mass Index (BMI) >30 Kg/m^2^:

$$ B S A\ \left({\mathrm{m}}^2\right) = \mathrm{square}\ \mathrm{root}\ \mathrm{of}\ \left( height\ \left(\mathrm{cm}\right)\kern0.5em \mathrm{x}\kern0.5em  weight\ \left(\mathrm{kg}\right)/3600\right) $$

Rituximab will be administered by intravenous infusion at a dose of 1000 mg. The dose will be repeated at 14 days. This second dose may be delayed by up to 10 days. If it is not given within this time it should be omitted.

Placebo infusions will be administered in order to maintain the blind and all patients will receive seven infusions in total.

Both cyclophosphamide and rituximab as well as the placebo will have identical appearances (clear, colourless liquid) and will be drawn up in identical volumes so as to avoid unblinding of the study drug.

### Non-Investigational Medicinal Product (nIMP) drugs

The following nIMPS will be used in this study and administered to both groups when they receive cyclophosphamide/rituximab/placebo. All nIMPS are open label and generic forms can be used:

1. Mesna will be administered to patients in both groups at day 0 and monthly until week 20. Mesna 200 mg will be given by intravenous infusion in 100 ml 0.9% sodium chloride over 30 min immediately prior to cyclophosphamide/rituximab/placebo. Additionally, Mesna 400 mg will be administered per os at 2 h and 400 mg at 6 h post cyclophosphamide/rituximab/placebo infusion
2. Hydrocortisone 100 mg by intravenous injection to be given 30 min prior to cyclophosphamide/rituximab/placebo at day 0 and day 14
3. Chlorphenamine 10 mg by intravenous injection to be given 30 min prior cyclophosphamide/rituximab/placebo at day 0 and day 14
4. Paracetamol 1 g to be given per os 30 min prior to cyclophosphamide/rituximab/placebo at day 0 and day 14
5. Patients will be offered ondansetron an antiemetic as required prior to, and for up to 3 days, following the cyclophosphamide/rituximab/placebo infusions

### Concomitant medication

Corticosteroids are frequently used for patients with CTD and it is anticipated that the majority of subjects will be taking concomitant steroid therapy. The choice of dose will rest with the subject-treating clinician but all changes in corticosteroids will be documented in the electronic Case Report Form (eCRF) to permit calculation of cumulative exposure. As general guidance it is anticipated that:

- Patients with scleroderma, corticosteroids will be maintained at a stable dose of prednisolone ≤10 mg daily
- Individuals with inflammatory myositis will require high-dose orally or intravenously administered corticosteroids at initiation of therapy but that these should be weaned to ≤20 mg prednisolone daily by treatment week 12
- Patients with MCTD should be maintained on a stable dose of ≤20 mg prednisolone daily following entry into the study

### Immunosuppressants

- At week 24, following completion of the treatment phase of the study and after measurement of the primary endpoint, patients will be permitted to commence additional immunosuppressant therapy according to the recommendations of their treating physician
- All other disease-specific, nonimmunosuppressant, therapies will be permitted for the duration of the study. Patients may also receive n-acetylcysteine up to 600 mg three times daily (t.d.s.)

### Prohibited concomitant medication

- Pre-existing immunosuppression (including azathioprine, mycophenolate mofetil, methotrexate and cyclosporine), as is standard practice prior to cyclophosphamide administration, will be stopped at least 14 days prior to randomisation
- Between weeks 0–24, patients will not be permitted to receive additional immunosuppression (including orally administered agents, intravenously administered immunoglobulins or other monoclonal antibody therapies) other than corticosteroids

## Outcomes

### Primary outcome measure

- Absolute rate of change in forced vital capacity (FVC) at week 24

### Secondary outcome measures

- Change from baseline in diffusing capacity for carbon monoxide (DLco) at 24 weeks
- Change from baseline in health-related quality of life scores (St. George’s Respiratory Questionnaire (SGRQ), Short form (36) Health Questionnaire (SF-36), King’s Brief Interstitial Lung Disease (K-BILD))
- Change from baseline in global disease activity score
- Change in 6-min walk test distance over 48 weeks
- Change in FVC and DLco at 48 weeks
- Further analyses on FVC
  - ○ Absolute categorical change of %FVC at 24 and 48 weeks (decrease by >5%, increase by >5% and change within <5%)
  - ○ Absolute categorical change of %FVC at 24 and 48 weeks (decrease by >10%, increase by >10% and change within <10%)
  - ○ 48-week rate of change in FVC
- Disease-related mortality (adjudicated by steering committee at close of study)
- Overall survival
- Progression-free survival (composite endpoint of mortality, transplant, treatment failure or decline in FVC >10% compared to baseline)
- Treatment failure (as determined by need for transplant or rescue therapy with either open-label cyclophosphamide or rituximab at any point until 48 weeks)
- Total corticosteroid requirement over 48 weeks
- Change from baseline in capillary oxygen saturation (SpO_2_) at 24 and 48 weeks
- Health care utilisation during study period (visits to primary care, unscheduled hospital visits, emergency admissions)
- Scleroderma-specific endpoints (change in Scleroderma Health Assessment Questionnaire (Scleroderma HAQ), modified Rodnan Skin Score (mRSS)).
- Safety and tolerability

### Exploratory outcome measures

- Change in lymphocyte subsets relative to outcome in the rituximab group
- Change in plasma cytokine levels following therapy and in relationship to markers of disease activity (FVC, DLco, quality of life, global disease activity scores)
- Change in candidate serum biomarkers of fibrosis (to include KL-6, MMP-1, MMP-7, Sp-A and Sp-D) following therapy
- Outcome in relation to underlying CTD

### Participant timeline

**Fig. 2:**
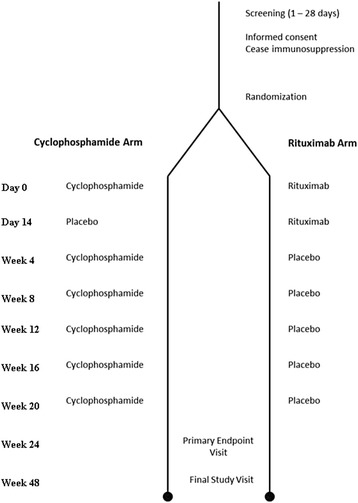
Flowchart of study design

The Standard Protocol Items: Recommendations for Interventional Trials (SPIRIT) schedule of events for the enrolment, interventions and assessments for participants is shown in Figs. 1 and 2. An indexed SPIRIT Checklist can be found in Additional file 2.

### Assignment of interventions

Randomisation allocation will be released using an Interactive Web-based Randomisation System (IWRS: InForm). Patients will be randomised (in a 1:1 double-blind fashion) to receive rituximab or cyclophosphamide. To ensure an equal representation of CTD subtypes in each treatment arm, randomisation will be stratified based upon underlying CTD diagnosis (according to the three diagnostic categories listed in the inclusion criteria of the study protocol). Access to the IWRS at each participating centre will be restricted to authorised study staff.

### Sample size estimate

Previous studies of intravenously administered cyclophosphamide in SSc demonstrated a 1% decline in FVC at 12 months, with a coefficient of variation of 7.8% [6, 7]. Our observational data and a previous nonrandomised study of rituximab (used as rescue therapy in those failing treatment with cyclophosphamide) suggest improvements in FVC at 6–12 months of between 9.5 and 20% compared to baseline [19]. Using 1:1 randomisation, a sample size of 52 patients in each group will have a 90% power to detect a 5% difference (approximately 140 ml) between groups at 24 weeks in the change in FVC (as measured in millilitres) with a significance level (alpha) of 0.05 (two-tailed). Anticipating a dropout rate of 10% our target recruitment is, therefore, 58 patients in each arm of the study. On the basis of data derived in other ILD studies, a 5% change in FVC is associated with change in long-term prognosis and can, therefore, be considered a clinically meaningful difference between the two groups [26]. Given the number of individuals treated with cyclophosphamide at our unit and in units that will be participating in the study, this number is feasible to deliver within the planned trial timelines.

### Data collection and management

The primary efficacy measurement will be change in FVC. Centres will be asked to undertake clinical trial spirometry on a single, specified spirometer within their clinical physiology department or clinical trials unit. Spirometry will be undertaken by a named individual or individuals who have had training to the standard recommended by the Association for Respiratory Technology and Physiology (ARTP). Usage of spirometers must meet the standards outlined in the American Thoracic Society (ATS)/European Respiratory Society (ERS) guidelines (P05-12782), including daily calibration of the spirometer, and regular calibration of the calibration pump. Spirometry will be conducted whilst the patient is in a seated position. The test will be done in triplicate and selection of the best result done according to the guidelines. Spirometric results will be filed in the local medical records and will be available for review as required.

For each patient, pulmonary function testing will always start at approximately the same time of the day (with ±60 min maximum difference, time will be recorded). On days of clinic visits (including the screening visit), patients must refrain from strenuous activity for at least 12 h prior to pulmonary function testing.

Smoking will be discouraged throughout the study day (clinic visit) and will not be permitted in the 30-min period prior to spirometry. Patients should also avoid cold temperatures, environmental smoke, dust, or areas with strong odours (e.g. perfumes). Washout of bronchodilator therapies (beta-agonist or anticholinergic drugs) should be undertaken before spirometry: 24 h for long-acting and 8 h for short-acting bronchodilators.

### Assessment of safety

The checking for the occurrence of adverse events (AEs) and clinical endpoints will begin from randomisation and will continue for the individual patient until they complete their follow-up at 48 weeks. At each study visit the investigator or designee will make an assessment of safety and will specifically review the clinical history and investigation findings with regard to the occurrence of adverse or serious adverse events (SAEs). Details of adverse and clinical events will be captured on the trial eCRF.

### Research blood samples

All biological samples for future research will be collected and handled according to a study-specific procedure, stored anonymously and labelled using a unique study number to permit accurate linkage to clinical data. Samples will be initially processed and stored at study sites in accordance with the study-specific procedure for handling RECITAL biological samples, to facilitate transfer to the Royal Brompton Hospital (RBH) Biological Research Unit (BRU) Royal Brompton Hospital (RBH), Sydney Street, London, SW3 6NP.

The storage of samples and use in future unspecified research will be performed in accordance with the Human Tissue Act 2004, and the RBH policy for ‘the acquisition, storage and use of human biological specimens for research’. Stored samples may be used to assess future biomarkers for the risk of developing lung fibrosis and the prognosis of this condition.

### Routine safety blood tests

Routine safety blood tests including full blood count, urea and electrolytes and liver function tests will be undertaken in the clinical laboratories at local sites according to local policies and procedures.

### Quality of life assessment

Quality of life will be assessed by self-administered questionnaires. These will be completed at baseline and repeated at the first follow-up visit for primary endpoint at 24 weeks and final follow-up visit at 48 weeks. The instruments used will be the SF-36 and the EuroQol 5 dimensions health survey (EQ5D), the SGRQ, K-BILD and the Scleroderma Health Assessment Questionnaire.

### Health economics

The RECITAL study will provide reliable data about the efficacy and safety of rituximab compared to standard therapy. As rituximab is considerably more expensive than cyclophosphamide, the issue of cost-effectiveness and affordability will arise if we show that it is more effective than standard treatments. In a study of this size it may be difficult to perform standard cost-effectiveness analyses (e.g. cost per quality-adjusted life year (QALY)), but we can estimate cost-effectiveness using surrogate clinical outcomes and also reliably estimate costs for care in the two treatment groups including costs of drug, tests and investigations, health care visits (hospital, GP, clinic). We will also have a range of patient-based outcomes using validated questionnaires. Working with the health economic team at the University of East Anglia, the health economic analysis will deliver a high-quality analysis using standard techniques to inform our understanding of the cost-effectiveness of the new treatment.

### Discontinuation or withdrawal of study subjects

The study drug will be discontinued if, in the opinion of the local investigator/caring physician, an individual participant’s disease has progressed despite receiving study therapy. The decision regarding progression will rest with the local physician, but indicators of disease progression will include: worsening symptoms, progression of radiological changes and reduction in FVC of >10% or DLco of >15% from baseline or reduction in resting oxygen saturations from baseline. Individuals with progressive disease will be unblinded from the study and, if felt appropriate by their caring physician, may be offered the alternative treatment regimen on an open-label basis (i.e. patients receiving rituximab will be offered cyclophosphamide and those receiving cyclophosphamide will be considered for rituximab). Similarly, in the case of individuals discontinuing treatment because of AEs the option will be open to the local caring physician to initiate open-label treatment with the alternate treatment regimen.

Subjects discontinuing the study drug will be invited to continue with planned monitoring and end-of-study visits. Complete protocol required data will, whenever possible, be collected for all individuals who are randomised into the study whether or not they receive their assigned treatment or discontinue the study prematurely. Subjects discontinuing study treatment will be asked to return for the primary endpoint (week 24) and final (week 48) follow-up visits. Apart from the treatment week-12 visit they will not be required to attend any further treatment phase visits once treatment is discontinued.

### Permanent discontinuation of IMP

Permanent discontinuation of study medication should occur in the following circumstances:

- Consent withdrawn
- Pregnancy
- New diagnosis of tuberculosis, infective hepatitis or HIV

### Possible temporary discontinuation of IMP

In the following cases withdrawal of study drug is highly recommended:

- Episode of severe infection requiring prolonged antibiotic treatment (>14 days) or hospitalisation
- New diagnosis of bladder cancer
- New occurrence of neurological symptoms suggesting a diagnosis of progressive multifocal leukoencephalopathy (PML)

However, in special circumstances and after review of the clinical data, consultation with the appropriate specialist (urologist, neurologist) and members of the multidisciplinary team, an appropriate risk benefit assessment and consultation with the patient, the investigator may decide not to withdraw the subject. In each case, as this is an intention-to-treat trial, patients will be invited to continue attending study visits to allow for full collection of study data.

### Serious adverse event (SAE) reporting and adverse event (AE) reporting

AEs and SAEs will be identified according to standard criteria and will be recorded in the eCRF and reported to the sponsor. Given the nature of participants’ underlying disease and also the known profile of the drugs under investigation, a number of expected AEs and SAEs have been defined. Expected adverse reactions are listed as the known side effects of the IMP reported in the Summary of Product Characteristics (SmPCs) of cyclophosphamide, rituximab and sodium chloride. Events that are expected and related to underlying disease will include study endpoints and disease progression or worsening of pre-existing respiratory or rheumatological symptoms.

### Data management and data checking

Data will be collected on an eCRF system. The InForm system will be used to develop the eCRF and will be designed in accordance with the requirements of the clinical trial protocol and will comply with regulatory requirements. Local personnel will be trained on the InForm system. Access will be restricted to site personnel, trial managers, trial monitors and the data management team. Personnel will have individual logon and passwords. It will be the investigator’s responsibility to ensure the accuracy of all data entered and recorded in the eCRFs. Trial monitors will check the accuracy of the eCRF data against source documents.

It is anticipated that the majority of source data (medical progress notes and letters, tests and investigations) will be filed in the individual patients’ medical records. Any deviation from source data being present in the medical notes will be identified and documented. The eCRF and source documents must be available at all times for review by the sponsor’s clinical trial monitor, auditors and for inspection by the Medicines Health Regulatory Agency. The accuracy of eCRF data will be verified by review of the source documents and details will be provided in the Trial Monitoring Report.

### Statistical analysis

Before starting the data analysis, the level and pattern of the missing data in the baseline variables and outcomes, and any treatment group crossovers, will be established by forming appropriate tables. The likely causes of any missingness and crossovers will be investigated. This information will be used to determine whether the level and type of missing data and the crossover rate have the potential to introduce bias into the analysis results for the proposed statistical methods, or substantially reduce the precision of estimates related to treatment effects.

### Primary efficacy analysis

- Analysis of the primary outcome will be by intention-to-treat. The data will be analysed according to the initial randomisation groups with no changes made in respect of subsequent withdrawals or crossovers
- The hypothesis to be tested is that rituximab is superior to cyclophosphamide. The study will be considered positive if statistical significance at the level of 0.05 (two-tailed) is achieved
- To test the hypothesis above and estimate the difference in FVC at week 24 and its 95% confidence interval, a three-level hierarchical (mixed/multilevel) model will be used: let *FVC*_*iw*_ represent the FVC (in millilitres) for patient *i* at week *w* and *t* (*i*) represent the treatment given to individual *i* (rituximab or cyclophosphamide). So, we model *FVC*_*iw*_ as the sum of four components:
  $$ D{S}_{i w}= intercep{t}_i+ change\kern0.75em  over\kern0.75em  t i m{e}_{t(i) w}+ C T{D}_i+ residual\kern0.75em  erro{r}_{i d} $$
- Intercept term: represents the estimate FVC on week 0 (the start of the treatment, first visit after randomisation). This term will comprise an individual-level random effect which will be drawn from a distribution parameterised using the associated centre-level random effect. Hence, the unexplained variation in the FVC scores will be split in to three components corresponding to the three levels of the model, i.e. the variation attributable to the centre (between-centre variation) and the individual (between-individual variation), as well as the residual variation (within-individual variation). CTD diagnosis stratum (categorical) used for randomisation will be added as a covariate. Other baseline covariates might be added if further analysis reveals a substantial imbalance
- Change over time term: this represents a coefficient which captures the changes in FVC over time (measured in weeks) and an interaction term between time and treatment. This interaction term will capture the difference in change of FVC between the two treatment groups per week. The magnitude at 24 weeks and its 95% confidence interval will be calculated to answer the research question.

Linear change is assumed over time with different slopes (the interaction term represents the difference in the slope); however, alternatives will be considered if the rate of change is not constant over the 24-week period. Alternatives are to include quadratic and square root terms. This will be assessed before the unblinding.

Residual error term: it is assumed that the residual errors have a normal distribution.

### Secondary efficacy analysis

- Analysis of secondary efficacy outcomes will also be by intention-to-treat
- Change in continuous physiological variables between baseline and 48 weeks will be assessed by similar multilevel modelling as described for the primary outcome
- Categorical change in physiological variables will be measured using chi-squared tests under the null hypothesis of no difference between the treatment groups
- Mortality, treatment failure and progression-free survival will be measured using Kaplan-Meier estimates. A log-rank test will be used to compare treatment groups and a Cox proportional hazards model will be used to determine hazard ratios for survival analyses

### Interim analyses

No formal interim analysis is planned. A regular review of safety data will be conducted to monitor the safety of patients in the trial. A Data Monitoring Committee (DMC) will follow number of deaths, early discontinuation due to AEs and SAEs in an unblinded fashion. A planned DMC meeting will be held to review all available data after the 12th randomised patient has completed the week-24 visit and twice yearly.

### End of study

The primary endpoint for the trial is at 24 weeks, with a final study visit timed at 48 weeks to determine longer-term efficacy and to ensure that all significant AEs are detected. The trial will formally end when the final subject completes their week-48 visit.

### Ethics and dissemination

The study conduct will comply with all relevant laws of the EU and all relevant laws and statutes of the UK including, but not limited to, the Human Rights Act 1998, the Data Protection Act 1998, the Medicines Act 1968, the Medicines for Human Use (Clinical Trial) Regulations 2004, and with all relevant guidance relating to medicines and clinical studies from time to time in force including, but not limited to, the ICH GCP, the World Medical Association Declaration of Helsinki entitled ‘Ethical Principles for Medical Research Involving Human Patients’ (2008 Version), the NHS Research Governance Framework for Health and Social Care (Version 2, April 2005).

The study was approved on 18 October 2013 by the UK Medicines and Healthcare Regulatory Agency in compliance with the European Clinical Trials Directive and the Medicines for Human Use (Clinical Trials) Regulations 2004 and its subsequent amendments. The study was provided with ethical approval by the NRES Committee London Westminster (Ref no.: 13/LO/0968) on 20 August 2013. The approved patient information sheet and consent form are available in Additional files 3 and 4.

Data from the study will be published in abstract form at international meetings and will be submitted for publication in a peer-reviewed journal and will be reported to the study funder.

## Discussion

ILD is an important cause of morbidity and mortality in patients with CTD. This trial should better define the optimal management of patients with severe CTD-ILD. Current therapy is limited by side effects and the risk of infection associated with significant immunosuppression. Although rituximab costs more on an individual dose basis, it necessitates fewer visits for administration and may be more convenient for patients and less expensive overall.

Additionally, the RECITAL study will evaluate several novel biomarkers for their ability to predict disease behaviour and response to therapy in CTD-ILD. These biomarkers have already been demonstrated to reflect fibrotic activity in various ILDs and may, therefore, be of value in detecting patients at risk of rapid progression of disease who require aggressive immunomodulation.

### Trial status

Recruitment to RECITAL began in November 2014. The study is currently actively recruiting in the UK.

## Additional files

Additional file 1: Protocol V6.1_15.12.14. (DOC 787 kb)

Additional file 2: SPIRIT Checklist. (DOC 125 kb)

Additional file 3: Patient Information Sheet. Version 6.0 15.10.2014. (DOC 217 kb)

Additional file 4: Informed Consent Form. Version 6.0. 15.10.2014. (DOC 39 kb)

Additional file 5: Exclusion criteria. (DOCX 90 kb)

## Abbreviations

CTD: Connective tissue disease
DLco: Diffusing capacity of the lung for carbon monoxide
FVC: Forced vital capacity
ILD: Interstitial lung disease
NSIP: Nonspecific interstitial pneumonia
SF-36: Short form (36) Health Questionnaire
SGRQ: St. George’s Respiratory Questionnaire
UIP: Usual interstitial pneumonia
