# Supplementary material for: Rituximab versus cyclophosphamide for the treatment of connective tissue disease-associated interstitial lung disease (RECITAL): study protocol for a randomised controlled trial
Source: Trials. 2017 Jun 15;18:275. doi: 10.1186/s13063-017-2016-2 (PMC5471887; doi:10.1186/s13063-017-2016-2)
Supplement: Supplementary file 1 — Protocol V6.1_15.12.14. (DOC 787 kb) [file 13063_2017_2016_MOESM1_ESM.doc]

|  |
| --- |
| A randomized, double blind controlled trial comparing **R**ituximab against intrav**e**nous **C**yclophosphamide in Connective Tissue Disease (CTD) associated **I**nters**t**iti**a**l **L**ung Disease (ILD) |
| **RECITAL** |
| Protocol version 6.1, 15^th^ December 2014 |
|  |
| **Sponsor’s R&D Registration Number:** 2012OE003B |
| **EudraCT number:** 2012-003633-42 |
| **Chief Investigator:** Toby Maher |
|  |

**Signature Page**

The Chief Investigator (CI) and the Royal Brompton and Harefield NHS Foundation Trust Research Office have discussed this protocol. The investigator agreed to perform the investigations and to abide by this protocol except in the case of medical emergency or where departures from it are mutually agreed in writing.

The Investigator agrees to conduct the trial in compliance with the protocol, GCP and UK Regulations for CTIMPs, the Data Protection Act (1998), the Trust Information Governance Policy (or other local equivalent), the Research Governance Framework (2005), SOPs agreed with the study Sponsor, and other regulatory requirements as appropriate.

| **Chief Investigator**  Dr Toby Maher  Consultant Respiratory Physician |  |  |
| --- | --- | --- |
| Royal Brompton and Harefield NHS Foundation Trust | Signature | Date |
| **Sponsor Representative**  Dr Angela Cooper  Associate Director of Research |  |  |
| Royal Brompton and Harefield NHS Foundation Trust | Signature | Date |

CONTACTS

| **Sponsor:** | Royal Brompton and Harefield NHS Foundation Trust (RB&HFT) |
| --- | --- |
| **Sponsor Contact:** | Dr Angela Cooper  Research Office  Sydney Street  London, SW3 6NP  Email: [a.cooper@rbht.nhs.uk](mailto:a.cooper@rbht.nhs.uk) |
| **Chief Investigator:**  **Medical Contact:** | Dr Toby Maher  Interstitial Lung Disease Unit  Royal Brompton Hospital  Sydney Street, London, SW3 6NP.  Telephone No.: 020 7352 8121 x 4188  Mobile No.: 07971 269 262  [t.maher@rbht.nhs.uk](mailto:t.maher@rbht.nhs.uk) |
| **Co-Investigators:** | Dr Greg Keir [G.Keir@rbht.nhs.uk](mailto:G.Keir@rbht.nhs.uk)  Prof Chris Denton [c.denton@ucl.ac.uk](mailto:c.denton@ucl.ac.uk)  Prof Athol Wells [Athol.wells@rbht.nhs.uk](mailto:Athol.wells@rbht.nhs.uk)  Dr Helen Parfrey [hp226@cam.ac.uk](mailto:hp226@cam.ac.uk)  Prof Marcus Flather [m.flather@uea.ac.uk](mailto:m.flather@uea.ac.uk)  Prof John Pepper [j.pepper@rbht.nhs.uk](mailto:j.pepper@rbht.nhs.uk)  Prof Deborah Ashby [deborah.ashby@imperial.ac.uk](mailto:deborah.ashby@imperial.ac.uk) |
| **Trial Management:** | Imperial Clinical Trials Unit (ICTU)  Imperial College London  ICCH Building, 59-61 North Wharf Road  London, W2 1PG  RECITAL Trial Manager  Interstitial Lung Disease Unit  Royal Brompton and Harefield NHS Foundation Trust  Royal Brompton Hospital  Sydney Street, London, SW3 6NP  Tel: 020 7352 8121 |
| **Blood Samples Laboratory** | Dr Toby Maher  Biological Research Unit  Royal Brompton Hospital  Sydney Street, London, SW3 6NP.  Telephone No: 020 7352 8121 x 4188 |

CONTENTS

[1 Study Synopsis 6](#__RefHeading___Toc401307204)

[2 Study Flow Diagram 8](#__RefHeading___Toc401307205)

[3 introduction 10](#__RefHeading___Toc401307206)

[3.1 Background 10](#__RefHeading___Toc401307207)

[3.2 Existing Research 10](#__RefHeading___Toc401307208)

[3.3 Risks and Benefits 11](#__RefHeading___Toc401307209)

[3.4 Rational for Current Study 12](#__RefHeading___Toc401307210)

[4 Objectives 12](#__RefHeading___Toc401307211)

[4.1 Primary Objective 12](#__RefHeading___Toc401307212)

[4.2 Secondary Objectives 12](#__RefHeading___Toc401307213)

[5 Study Description 13](#__RefHeading___Toc401307214)

[5.1 Study Design 13](#__RefHeading___Toc401307215)

[5.2 Treatment Regimens 13](#__RefHeading___Toc401307216)

[Rituximab group 13](#__RefHeading___Toc401307217)

[Cyclophosphamide group 13](#__RefHeading___Toc401307218)

[5.3 Study Population 14](#__RefHeading___Toc401307219)

[5.4 Biomarkers 14](#__RefHeading___Toc401307220)

[6 Study Eligibility Criteria 15](#__RefHeading___Toc401307221)

[6.1 Inclusion Criteria 15](#__RefHeading___Toc401307222)

[6.2 Exclusion Criteria 15](#__RefHeading___Toc401307223)

[7 Study Procedures 16](#__RefHeading___Toc401307224)

[7.1 Screening and identification of patients 16](#__RefHeading___Toc401307225)

[7.2 Informed Consent 16](#__RefHeading___Toc401307226)

[7.3 Blinding 17](#__RefHeading___Toc401307227)

[7.4 Randomisation 18](#__RefHeading___Toc401307228)

[7.5 Patient Emergency Study Contact Card 18](#__RefHeading___Toc401307229)

[7.6 Emergency Un-blinding Procedure 18](#__RefHeading___Toc401307230)

[8 trial observations, tests and Investigations 19](#__RefHeading___Toc401307231)

[8.1 Timing of Assessments 19](#__RefHeading___Toc401307232)

[8.2 Screening Assessment 19](#__RefHeading___Toc401307233)

[8.3 Baseline and First Treatment 19](#__RefHeading___Toc401307234)

[8.4 Treatment Visits (weeks 2/4/8) 20](#__RefHeading___Toc401307235)

[8.5 Treatment Visit (week 12) 21](#__RefHeading___Toc401307236)

[8.6 Treatment Visit (weeks 16 and 20) 22](#__RefHeading___Toc401307237)

[8.7 Primary End-Point Assessment (Week 24) 22](#__RefHeading___Toc401307238)

[8.8 Final Follow-up Assessment (week 48) 23](#__RefHeading___Toc401307239)

[8.9 Unscheduled Visits 24](#__RefHeading___Toc401307240)

[8.10 Assessment of Efficacy 26](#__RefHeading___Toc401307241)

[8.11 Assessment of Safety 26](#__RefHeading___Toc401307242)

[8.12 Research Blood Samples 27](#__RefHeading___Toc401307243)

[8.13 Routine Safety Blood Tests 27](#__RefHeading___Toc401307244)

[8.14 Other Routine Laboratory Tests 27](#__RefHeading___Toc401307245)

[8.15 Quality of Life Assessment 28](#__RefHeading___Toc401307246)

[9 Treatment 28](#__RefHeading___Toc401307247)

[9.1 Name and description of each IMP 28](#__RefHeading___Toc401307248)

[9.2 Source of IMPs including placebo 28](#__RefHeading___Toc401307249)

[9.3 Labelling 28](#__RefHeading___Toc401307250)

[9.4 Storage and Administration 28](#__RefHeading___Toc401307251)

[9.5 Accountability procedures for the IMP(s) 29](#__RefHeading___Toc401307252)

[9.6 Route of Administration, Dosage and Treatment Period(s) of the IMPs 29](#__RefHeading___Toc401307253)

[9.7 Dosage Modifications 30](#__RefHeading___Toc401307254)

[9.8 Assessment of Compliance 30](#__RefHeading___Toc401307255)

[9.9 Post-trial IMP Arrangements 30](#__RefHeading___Toc401307256)

[9.10 Permitted Concomitant Medication 30](#__RefHeading___Toc401307257)

[9.10.1 Non-IMP (nIMP) 30](#__RefHeading___Toc401307258)

[9.10.2 Corticosteroids 31](#__RefHeading___Toc401307259)

[9.10.3 Immunosuppressants 31](#__RefHeading___Toc401307260)

[9.11 Prohibited Concomitant Medication 31](#__RefHeading___Toc401307261)

[10 Patient withdrawal 32](#__RefHeading___Toc401307262)

[10.1 Discontinuation of IMP Due to Progression of Underlying Disease 32](#__RefHeading___Toc401307263)

[10.2 Permanent Discontinuation of IMP 32](#__RefHeading___Toc401307264)

[10.3 Possible Temporary Discontinuation of IMP 32](#__RefHeading___Toc401307265)

[10.4 Withdrawal from Trial Procedures and Incomplete Follow-up 33](#__RefHeading___Toc401307266)

[11 Endpoints 33](#__RefHeading___Toc401307267)

[11.1 Primary Endpoint 33](#__RefHeading___Toc401307268)

[11.2 Secondary Endpoints 33](#__RefHeading___Toc401307269)

[11.3 Safety and Tolerability Endpoints 34](#__RefHeading___Toc401307270)

[11.4 Exploratory endpoints 34](#__RefHeading___Toc401307271)

[11.5 Health economics 35](#__RefHeading___Toc401307272)

[12 pharmacovigilAnce 35](#__RefHeading___Toc401307273)

[12.1 Pharmacovigilance and Safety Reporting 35](#__RefHeading___Toc401307274)

[12.2 Definitions 35](#__RefHeading___Toc401307275)

[12.2.1 Adverse Event (AE) Definition 35](#__RefHeading___Toc401307276)

[12.3 Adverse Reaction (AR) Definition 36](#__RefHeading___Toc401307277)

[12.4 Serious Adverse Event (SAE) or Serious Adverse Reaction (SAR) 36](#__RefHeading___Toc401307278)

[12.5 Suspected Unexpected Serious Adverse Reaction (SUSAR) 36](#__RefHeading___Toc401307279)

[12.6 Expedited Reporting of SUSARs 37](#__RefHeading___Toc401307280)

[12.7 Reporting Adverse Events (AEs) – (including SAE/Rs) 37](#__RefHeading___Toc401307281)

[12.8 Expected SAE/Rs 37](#__RefHeading___Toc401307282)

[12.8.1 Expected SAE/Rs Related to IMP 37](#__RefHeading___Toc401307283)

[12.8.2 Expected SAEs Related to Underlying Disease 38](#__RefHeading___Toc401307284)

[12.9 Severity of Adverse Events 38](#__RefHeading___Toc401307285)

[12.10 Causality / Relationship to Treatment 38](#__RefHeading___Toc401307286)

[12.11 The type and duration of the follow-up of patients after AEs 39](#__RefHeading___Toc401307287)

[12.11.1 Development Safety Update Reports (DSURs) 39](#__RefHeading___Toc401307288)

[12.11.2 Annual Progress Reports (APRs) 39](#__RefHeading___Toc401307289)

[12.12 Pregnancy reporting 39](#__RefHeading___Toc401307290)

[12.13 Safety Reporting Requirements for nIMPS 39](#__RefHeading___Toc401307291)

[12.14 Reporting Urgent Safety Measures 40](#__RefHeading___Toc401307292)

[12.15 Notification of Serious Breaches of GCP 40](#__RefHeading___Toc401307293)

[13 Data Management 40](#__RefHeading___Toc401307294)

[13.1 Data Collection 40](#__RefHeading___Toc401307295)

[13.2 Source Data 40](#__RefHeading___Toc401307296)

[13.3 Archiving 41](#__RefHeading___Toc401307297)

[14 Statistical issues 41](#__RefHeading___Toc401307298)

[14.1 Sample Size 41](#__RefHeading___Toc401307299)

[14.2 Statistical Analysis Plan 42](#__RefHeading___Toc401307300)

[14.3 Primary Outcome 42](#__RefHeading___Toc401307301)

[14.4 Secondary Outcomes 42](#__RefHeading___Toc401307302)

[14.5 Safety Analysis 43](#__RefHeading___Toc401307303)

[14.6 Interim analyses 43](#__RefHeading___Toc401307304)

[15 Trial Management and committees 44](#__RefHeading___Toc401307305)

[15.1 Trial Management and Structure 44](#__RefHeading___Toc401307306)

[15.2 Trial Steering Committee (TSC) 44](#__RefHeading___Toc401307307)

[15.3 Data Monitoring Committee (DMC) 44](#__RefHeading___Toc401307308)

[15.4 Trial Management 45](#__RefHeading___Toc401307309)

[15.5 Investigators’ Responsibilities 45](#__RefHeading___Toc401307310)

[15.6 Patient and Public Involvement 45](#__RefHeading___Toc401307311)

[16 regulatory, Ethical and legal issues 46](#__RefHeading___Toc401307312)

[16.1 Study Conduct 46](#__RefHeading___Toc401307313)

[16.2 Regulatory Requirements 46](#__RefHeading___Toc401307314)

[16.3 Ethical Requirements 46](#__RefHeading___Toc401307315)

[16.4 Institutional Approval 47](#__RefHeading___Toc401307316)

[16.5 Funding 47](#__RefHeading___Toc401307317)

[16.6 Insurance and Indemnity 47](#__RefHeading___Toc401307318)

[16.7 Confidentiality and Data Protection 47](#__RefHeading___Toc401307319)

[17 Quality Control and assurance 48](#__RefHeading___Toc401307320)

[17.1 Monitoring 48](#__RefHeading___Toc401307321)

[17.2 Audits and Inspections 48](#__RefHeading___Toc401307322)

[18 End of trial 49](#__RefHeading___Toc401307323)

[18.1 Planned Termination 49](#__RefHeading___Toc401307324)

[18.2 Premature Termination by Sponsor 49](#__RefHeading___Toc401307325)

[18.3 Premature Termination by the Investigator 50](#__RefHeading___Toc401307326)

[19 Data Ownership and Publication Policy 50](#__RefHeading___Toc401307327)

[20 References 51](#__RefHeading___Toc401307328)

[21 Appendix 1: Specific Adverse Events 53](#__RefHeading___Toc401307329)

# Study Synopsis

| **Title** | A randomized, double blind controlled trial comparing **R**ituximab against intrav**e**nous **C**yclophosphamide in Connective Tissue Disease (CTD) associated **I**n**t**erstiti**a**l **L**ung Disease (ILD) |
| --- | --- |
| **Acronym** | RECITAL Study |
| **Short Title** | Rituximab for CTD-ILD |
| **Chief Investigator** | Toby Maher |
| **Objectives** | The overall aims of this study are;  1) To demonstrate that intravenous Rituximab has superior efficacy to current best treatment (intravenous Cyclophosphamide) for CTD-ILD.  2) To compare the safety profile of Rituximab to intravenous Cyclophosphamide in individuals with CTD-ILD  3) To assess the health economic benefits of Rituximab compared to current standard of care for CTD-ILD  4) To evaluate a range of exploratory biomarkers for disease severity, prognosis and treatment response in CTD-ILD |
| **Study Design** | Randomized, double blind, double dummy, Phase 2b |
| **Setting** | UK multi-center study |
| **Sample Size Estimate** | Sample size: 116 (aiming for 52 patients reaching end-of-study in each arm and anticipating a 10% drop out)  Statistical analysis: Previous studies of IV Cyclophosphamide in systemic sclerosis showed a 1% decline in FVC at 12 months with a standard deviation of 7.8%. Our observational data and a previous non-randomised study of Rituximab suggest improvements in FVC at 6 months of up to 19%. Using a 1:1 randomization a sample size of 52 patients in each group will have a 90% power to detect a 5% difference in 24 week FVC between treatment groups with a significance level (alpha) of 0.05 (two-tailed). |
| **Number of Participants** | 116 |
| **Eligibility Criteria** | A CTD diagnosis of systemic sclerosis, idiopathic inflammatory myopathy, or mixed CTD (according to accepted international criteria), and associated ILD |
| **Description of Interventions** | **Rituximab group:**  Rituximab will be given at a dose of 1000 mg at day 0 and day 14. At week 4 through to week 20 patients will receive placebo.  **Cyclophosphamide group:**  Cyclophosphamide will be given at a dose of 600 mg/m^2^ body surface area every 4 weeks from day 0 through to week 20. At day 14 the group will receive placebo.  **Concomitant Medication**  Patients may be administered open label, prednisolone according to the recommendations of their treating physician |
| **End of Study** | Completion of the last follow-up visit for the last patient recruited. |
| **Randomization** | 1:1 randomization stratified by CTD diagnostic category |
| **Outcome Measures** | **Primary outcome:** Change in forced vital capacity (FVC) at 24 weeks.  **Secondary outcomes:** Safety, change in diffusing capacity for carbon monoxide (DLco), change in 6 minute walk distance, change in quality of life scores (as measured by SGRQ, SF-36), change in global disease activity, overall healthcare utilization and change in FVC at 48 weeks  Exploratory outcome measures: a priori sub group analysis, novel biomarker assessment |
| **Statistical Methods** | Analysis of the primary outcome will be by intention to treat. Analysis of covariance (ANCOVA), including baseline FVC, the stratification factors for randomization and treatment group will be used as independent variables for the analysis. The hypothesis to be tested is that Rituximab is superior to Cyclophosphamide. The study will be considered positive if statistical significance at the level of 0.05 (two tailed) is achieved. |

# Study Flow Diagram

**Day 0**

**Day 14**

**Week 8**

**Week 4**

**Week 12**

**Week 16**

**Week 20**

**Week 24**

**Week 48**

Randomisation

ABBREVIATIONS

| ADR | Adverse Drug Reaction |
| --- | --- |
| AE | Adverse Event |
| ANCA | Antineutrophil Cytoplasmic Antibody |
| BRU | Biological Research Unit |
| CI | Chief Investigator |
| COPD | Chronic obstructive pulmonary disease |
| CTD | Connective Tissue Disease |
| DLco | Diffusing capacity of the lung for carbon monoxide |
| DNA | Deoxyribonucleic acid |
| DSUR | Development safety update reports |
| ECG | Electrocardiogram |
| ESR | Erythrocyte Sedimentation Rate |
| FEC | Full Economic Cost |
| FVC | Forced Vital Capacity |
| HRCT | High Resolution Computer Tomography |
| ICTU | Imperial Clinical Trials Unit |
| ILD | Interstitial Lung Disease |
| IMP | Investigational Medicinal Product |
| LPLV | Last Patient Last Visit |
| MCTD | Mixed Connective Tissue Disease |
| NIMP | Non Investigational Medicinal Product |
| NSIP | Non-Specific Interstitial Pneumonia |
| PI | Principal Investigator |
| PML | Progressive Multifocal Leukoencephalopathy |
| QOL | Quality of Life |
| RBH | Royal Brompton Hospital |
| RNA | Ribonucleic Acid |
| SAE | Serious Adverse Event |
| SOPs | Standard Operating Procedure |
| TSC | Trial Steering Committee |
| TTP | Thrombocytopenic Purpura |

# introduction

## Background

Interstitial Lung Disease (ILD) is characterised by inflammation and/or fibrosis that results in thickening and distortion of the alveolar wall with consequent impairment of gas exchange. Affected individuals typically present with progressive breathlessness which frequently causes respiratory failure and death. There are many described causes of ILD, however, one of the commonest is that resulting from lung involvement by systemic autoimmune disease. This group of conditions, the Connective Tissue Diseases (CTD), are an important cause of disability and death in the working age population. Over the last decade improvements in therapy for the CTDs has seen the prognosis for individuals with these conditions dramatically improve. Despite these improvements in care there has been little, if any, change in therapy for ILD occurring as a consequence of CTD. For this reason for those individuals with CTD, respiratory disease has grown in importance. For many CTD sufferers disease-associated ILD is now the major cause of disability and exercise limitation whilst in systemic sclerosis it is now the principal cause of mortality in this patient group[^1^](#_ENREF_1).

The pathogenesis of CTD-ILD is complex and poorly understood. It is however, generally accepted that underlying immune system dysfunction and immune-mediated pulmonary inflammation are critical to CTD-ILD development and progression. Abnormalities of cellular and humoral immune function have been described in ILD associated with SSc[^2-4^](#_ENREF_2), idiopathic inflammatory myopathy[^5^](#_ENREF_5) and several other CTDs[^5^](#_ENREF_5). The mechanism by which these processes lead on to fibrosis remains poorly understood as do the factors that determine which individuals with CTD develop ILD. Nonetheless evidence from treatment trials suggest that modulation of inflammation with immunosuppressant therapies, particularly Cyclophosphamide, results in some regression of ILD and prevents the development of further fibrosis.

## Existing Research

Different CTDs manifest varying forms of ILD. Individuals with scleroderma and Mixed Connective Tissue Disease (MCTD) most commonly develop the histological lesion of non-specific interstitial pneumonia. Those with idiopathic inflammatory myositis typically have combined organising pneumonia and NSIP (referred to in the literature as fibrosing organising pneumonia). By contrast to these conditions, individuals with rheumatoid disease frequently have fibrosis with the histological pattern of usual interstitial pneumonia and tend to be resistant to therapy with high dose immunosuppression.

The field of rheumatology has seen rapid developments over the last decade with the introduction of a range of monoclonal antibody therapies that have revolutionised the standard of care for this patient group. Despite this there have been few if any improvements in the management of CTD associated ILD. Currently, standard of care for severe, progressive CTD-ILD includes immunosuppression with intravenous Cyclophosphamide (600 mg/m^2^) administered monthly for 6 months, followed by maintenance oral immunosuppression. Occasionally, this intensive immunosuppressive therapy fails to control pulmonary inflammation and alternative therapies may be required. Rituximab, a chimeric (human/mouse) monoclonal antibody with a high affinity for the CD20 surface antigen expressed on pre-B and B-lymphocytes, results in rapid depletion of B cells from the peripheral circulation for 6 to 9 months. Evidence for the effectiveness of B cell depletion exists in a number of immune-mediated conditions, including rheumatoid arthritis[^10-12^](#_ENREF_10), ANCA-associated vasculitis and immune Thrombocytopenic Purpura[^15^](#_ENREF_15) (TTP). Several case series suggest Rituximab may also be effective in ILD occurring in the context of immunological over-activity, with favourable responses reported in anti-synthetase (ASS) associated ILD[^16^](#_ENREF_16)) and SSc-ILD. Our own experience has demonstrated Rituximab to be an effective, potentially life-saving therapeutic intervention in the treatment of very severe, progressive CTD-ILD unresponsive to conventional immunosuppression[^19^](#_ENREF_19).

## Risks and Benefits

It is hoped that this study will advance the standard of care for individuals with CTD-ILD. Despite current best treatment, individuals with extensive ILD due to scleroderma have a median survival of less than 5 years and a similar poor prognosis is observed in individuals with inflammatory myositis and MCTD[^20^](#_ENREF_20). If Rituximab can be shown to improve six month and one year lung function in this group then it is to be hoped that this will translate in to improvements in longer term survival and associated reductions in morbidity. The simplified dosing regimen for Rituximab when compared to Cyclophosphamide also affords the potential for reducing the burden on patients (and their carers) of frequent hospital attendances. Similarly, although drug costs are higher for Rituximab, it is hoped that a Full Economic Costing (FEC) will demonstrate savings based on reduced utilisation of healthcare resources and fewer hospital visits. There are currently no available biomarkers for assessing response to therapy or risk of disease progression in CTD-ILD. By closely studying patients in each treatment arm and undertaking exploratory biomarker analysis it is hoped that we might identify potential disease and therapy specific biomarkers for future development and use in clinical practice.

Against the potential benefits must be balanced the risks of treatment. As noted, Rituximab is a well-established therapy for a range of indications and as such its safety profile is well known. Potential risks of therapy include; infusion reactions, infection, arthralgia and hypercholesterolaemia. Very rarely long term hypogammaglobulinaemia has been reported. These side effects can be balanced against those known to occur following Cyclophosphamide which include haemorrhagic cystitis, nausea and vomiting and in the longer term an increased incidence of bladder malignancy.

## Rational for Current Study

The aim of the study is to compare the safety and effectiveness of Rituximab against that of Cyclophosphamide as first line therapy in patients with severe, progressive Connective Tissue Disease (CTD) associated Interstitial Lung Disease (ILD). The study will therefore test the hypothesis that Rituximab is superior to intravenous Cyclophosphamide, in terms of both safety and efficacy, as a treatment for extensive and progressive Connective Tissue Disease associated Interstitial Lung Disease (CTD-ILD).

# Objectives

## Primary Objective

- To demonstrate that intravenous Rituximab has superior efficacy compared to current best treatment (intravenous Cyclophosphamide) for CTD-ILD. This will be measured by assessment of change in FVC at 24 weeks.

## Secondary Objectives

- To compare the safety profile of Rituximab to intravenous Cyclophosphamide in individuals with CTD-ILD
- To assess the health economic benefits of Rituximab compared to current standard of care for CTD-ILD – including measurements of healthcare utilisation, Quality of Life (QoL) and carer burden.
- To evaluate a range of exploratory biomarkers for disease severity, prognosis and treatment response in CTD-ILD.

# Study Description

## Study Design

The study is a UK multi-centre, prospective, randomised, double blind, double dummy trial of intravenous Rituximab compared with intravenous Cyclophosphamide in patients with severe, progressive CTD-ILD. Patients will be randomised to two group, both groups will receive placebo to match the different regimens.

## Treatment Regimens

### Rituximab group

Rituximab 1000mg will be given for two doses at day 0 and day 14.

Placebo will be administered monthly from week 4 to week 20

### Cyclophosphamide group

Cyclophosphamide will be given at a dose of 600 mg/m^2^ body surface area rounded to the nearest 100mg (BSA) every 4 weeks from day 0 through to week 20. Placebo will be given at day 14.

Body surface area should be calculated with baseline measurements using the Mosteller method:

BSA (m^2^) = square root of (height (cm) x weight (kg)/3600)

For dosage modifications in obese subjects (those with body mass index > 30), refer to the IMP Handling Manual.

**Table 1: Summary of Treatment G**roups

|  | **Rituximab group** | **Cyclophosphamide group** |
| --- | --- | --- |
| Day 0 | IV active rituximab 1000mg | IV 600mg mg/m^2^ body surface area |
| Day 14 | IV active rituximab 1000mg | Placebo |
| Week 4 | Placebo | IV 600mg mg/m^2^ body surface area |
| Week 8 | Placebo | IV 600mg mg/m^2^ body surface area |
| Week 12 | Placebo | IV 600mg mg/m^2^ body surface area |
| Week 16 | Placebo | IV 600mg mg/m^2^ body surface area |
| Week 20 | Placebo | IV 600mg mg/m^2^ body surface area |

## Study Population

Patients will be eligible for enrolment where the treating physician has made the decision on clinical grounds, to intervene with intravenous immunosuppression to minimize the risk of progressive lung damage as a result of associated defined CTD.

A total of 116 patients with confirmed Connective Tissue Disease (systemic sclerosis, idiopathic interstitial myopathy or mixed connective tissue disease) and associated Interstitial Lung Disease will be randomised with 58 in the Rituximab arm and 58 in the Cyclophosphamide arm. Patients will be followed up for 48 weeks.

## Biomarkers

Current clinical measures in ILD lack the sensitivity to determine, at any one point in time, individuals with progressive disease or those whom have failed to respond to therapy. Serum biomarkers therefore offer the attractive prospect of enhancing clinical monitoring of disease[^21^](#_ENREF_21). In CTD-ILD a number of inflammatory cytokines have been postulated to link with disease behaviour. Furthermore, measurement of CD-19 positive B lymophocytes provides a direct measure of the effectiveness of Rituximab in ablating the B cell population. It is not clear, in other disease settings, however, whether return of CD19 positive B-lymphoctes portends reactivation of disease. We will therefore monitor the relationship between lymphocyte subsets and therapeutic response in our study cohort.

In addition to using validated physiological measures of disease progression and response to therapy we will also collect serial samples of collect whole blood (for RNA and DNA analysis), serum and plasma to explore changes in the levels of a range of candidate biomarkers (to include, but not limited to, IL-6, IL-13, IL-17, Sp-A, KL-6 and MMP7). The aim of this part of the study will be to identify a candidate biomarker panel for use in future studies and for development as a clinical tool for guiding therapeutic decision making. Samples collected as part of this analysis will also be used (with appropriate informed consent) for future translational studies to explore pathogenetic mechanisms in CTD-ILD with a view to guiding future treatment development.

# Study Eligibility Criteria

## Inclusion Criteria

- A diagnosis of Connective Tissue Disease (CTD), based on internationally accepted criteria, in one of the following categories[^22-25^](#_ENREF_22):
  - Systemic sclerosis
  - Idiopathic interstitial myopathy (including polymyositis/dermatomyositis)
  - Mixed Connective Tissue Disease (MCTD)
- Severe and/or progressive Interstitial Lung Disease (ILD) associated with the underlying Connective Tissue Disease (CTD).
- Chest HRCT performed within 12 months of randomisation
- Intention of the caring physician to treat the ILD with intravenous Cyclophosphamide (with treatment indications including; deteriorating symptoms attributable to ILD, deteriorating lung function tests, worsening gas exchange or extent of ILD at first presentation) and where there is a reasonable expectation that immunosuppressive treatment will stabilize or improve CTD-ILD. In individuals with scleroderma it is anticipated that patients will fulfil the criteria for extensive disease defined by Goh *et al^19^*
- Written informed consent

## Exclusion Criteria

- Age <18 or >80 years.
- Previous treatment with Rituximab and/or intravenous Cyclophosphamide
- Known hypersensitivity to Rituximab or Cyclophosphamide or their components
- Significant (in the opinion of the investigator) other organ co-morbidity including cardiac, hepatic or renal impairment
- Co-existent obstructive pulmonary disease (*e.g.* asthma, COPD, emphysema) with pre bronchodilator FEV1/FVC < 70%
- Patients at significant risk for infectious complications following immunosuppression
  - Including HIV positive or other immunodeficiency syndromes (including hypogammaglobulineamia)
- Suspected or proven untreated tuberculosis
- Viral hepatitis
- Infection requiring antibiotic treatment in the preceding four weeks
- Unexplained neurological symptoms (which may be suggestive of progressive mutifocal leukoencephalopathy; PML). Neurological symptoms arising as a consequence of the underlying CTD do not necessitate exclusion.
- Other investigational therapy (participation in research trial) received within 8 weeks of randomisation
- Immunosuppressive or CTD disease modifying therapy (other than corticosteroids) received within 2 weeks of randomisation
- Pregnant or breast feeding women, or women of child-bearing potential, not using a reliable contraceptive method for up to 12 months following IMP
- Unexplained haematuria, or previous bladder carcinoma
- CT scan > 12 months from randomisation
- Unable to provide informed written consent

# Study Procedures

## Screening and identification of patients

Patients will be recruited prospectively from rheumatology or Interstitial Lung Disease (ILD) units at multiple UK centres. The diagnosis of CTD and associated ILD will be confirmed by the local study PI or Co-Investigator. Potential patients will be assessed for eligibility and approached for written informed consent. Patients will undergo a screening assessment (section 8.2) to ensure they meet all the eligibility criteria. A screening log will be kept of all patients with CTD-ILD considered for the study and will include date of birth, gender, date screened, reason not randomised.

## Informed Consent

The Principal Investigator (PI) or designees who have clinical responsibility for the care of the patients will be permitted to undertake informed consent. All individuals taking informed consent will be appropriately trained. Patients will be given an ethically approved patient information sheet and an appointment will be made for them with the research nurse or another member of the research team delegated the responsibility to discuss enrolment into the study. Consent to enter the study will be obtained after a full account has been provided of its nature, purpose, risks, burdens and potential benefits, and the patient has had the opportunity to deliberate. The patient will be allowed to specify the time they wish to spend deliberating, usually up to 24 hours. Periods shorter than 24 hours will be permitted if the patient feels that further deliberation will not lead to a change in their decision, and provided the person seeking consent is satisfied that the patient has fully retained, understood and deliberated on the information given. This provision has been made with the support of our patient advisory group. Likewise, periods longer than 24 hours will be permitted should the patient request this. The Investigator or designee will explain that the patients are under no obligation to enter the trial and that they can withdraw at any time during the trial, without having to give a reason.

The process for obtaining participant informed consent will be in accordance with the REC guidance, and Good Clinical Practice (GCP) and all other applicable regulatory requirements. The decision regarding participation in the study is entirely voluntary. The investigator or their designee shall emphasize to potential participants that consent regarding study participation may be withdrawn at any time without penalty or affecting the quality or quantity of their future medical care, or loss of benefits to which the participant is otherwise entitled. No study-specific interventions will be performed before informed consent has been obtained. All participants will provide written informed consent. The Informed Consent Form will be signed and dated by the participant before entering the study. The participant will receive a copy of the signed and dated consent form and the original will be retained with the other study data. A second copy will be filed in the participant’s medical notes and a signed and dated note made in the medical records that informed consent was obtained for the study.

If new safety information results in significant changes to the risk–benefit assessment, the consent form and the PIS will be reviewed and updated if necessary. Amended PIS need to be ethically approved before they can be implemented. All patients, including those already being treated, will be informed of the new information, given a copy of the revised consent form and asked to re-consent if they choose to continue in the study.

## Blinding

The study is double blind. Medication will be identified by a unique trial identifier. Packaging and labelling of the Intravenous infusions will be prepared according to Good Manufacturing Practice (GMP) regulations. Patients and investigators will remain blinded until after database lock and only the Data Monitoring Committee (DMC) will have access to un-blinded information.

## Randomisation

Randomisation allocation will be released using an Interactive Web-based Randomisation System (InForm). Patients will be randomised (in a 1:1 double blind fashion) to receive Rituximab or Cyclophosphamide. To ensure an equal representation of CTD subtypes in each treatment arm, randomisation will be stratified based upon underlying CTD diagnosis (according to the three diagnostic categories listed in the inclusion criteria (section 6.1 of the study protocol). Access to the IWRS at each participating centre will be restricted to authorised study staff.

## Patient Emergency Study Contact Card

Randomised patients will be given a study specific 24hrs emergency contact immediately following randomisation. The card will include: Study title, details of the IMPs, patient trial number, CI/PI’s contact details along with out of hours contact details in case of emergency.

## Emergency Un-blinding Procedure

This will be a double-blind study with both the patient and investigator blinded to study treatment. The investigator or treating physician may un-blind a patient’s treatment assignment in the case of an emergency, when knowledge of the study treatment is essential for the appropriate clinical management or welfare of the patient. The investigator will make the decision to un-blind and have 24 hour access to un-blinding the treatment assignment *via* the electronic database system (InForm). There will be an emergency manual backup system in case of electronic failure; each site will hold an emergency un-blinding code which will be held in a locked cupboard in pharmacy. If the InForm system is not accessible for technical reasons then the investigator will contact RB&HFT hospital pharmacist (and/or an on call pharmacist outside of working hours) *via* RB&HFT switchboard (Phone: 0207 352 8121) for the un-blinding of the treatment assignment. The investigator must notify the Sponsor as soon as possible. The date and reason for the un-blinding must be recorded in the appropriate data collection tool.

A patient will be withdrawn if their treatment code is un-blinded by the investigator or treating physician. The primary reason for discontinuation (the event or condition which led to the un-blinding) will be recorded in the eCRF.

# trial observations, tests and Investigations

## Timing of Assessments

The schedule for study visits including the assessments and procedures to be performed is presented in Table 2 Schedule of Events table. The scheduled study visits should occur at the specified week post-randomisation ± 7 days unless otherwise specified.

## Screening Assessment

The screening phase (minimum of 1 day, maximum of 28 days) is designed to establish baseline characteristics and, where necessary, to permit a minimum 14 day wash out period for patients receiving oral immunosuppressants (other than corticosteroids) prior to the decision to initiate intravenous therapy. If all inclusion and exclusion criteria are met at the end of the screening phase the patient will be randomised. If the patient is a screen failure because of active infection then the patient may be re-screened after appropriate treatment has been given. All reasons for screening failure will be documented. At the screening assessment the following assessments will be undertaken;

- Physical examination
- Vital signs
- Concomitant medication review
- Collection of blood for laboratory analyses (to include full blood count, ESR, urea and electrolytes, hepatitis B and C serology liver function tests and, if liver function tests are abnormal, hepatitis A)
- Urinalysis
- Pregnancy test (for women of child bearing potential)
- ECG
- Spirometry
- [6 Minute Walk Test](http://www.thoracic.org/statements/resources/pfet/sixminute.pdf)
- Lung function tests (plethysmography and gas transfer)

## Baseline and First Treatment

The investigator or designee will review the inclusion and exclusion criteria with the patient to confirm eligibility. Patients will then be randomized as described in Section 7.2. All randomized patients will undertake baseline investigations in the order outlined below and in the Schedule of Events prior to initiation of therapy. These include;

- Vital signs
- Concomitant medication review
- AE review prior to 1^st^ dose of IMP
- Collection of blood for laboratory analyses (to include full blood count, ESR, urea and electrolytes and liver function tests and, if liver function tests are abnormal, hepatitis serology)
- Blood for lymphocyte subsets
- Blood for RNA, DNA and Biomarker analysis
- Global Disease activity score
- Quality of Life Questionnaires and Health Economic Patient Diary
- Modified Rodnam’s Skin Score (scleroderma patients only)
- ECG
- Spirometry
- Lung Function (plethysmography and gas transfer)
- 6 Minute Walk Test
- Urinalysis

Once baseline blood test results are available and have been reviewed patients will be given the first dose of study treatment.

## Treatment Visits (weeks 2/4/8)

At each visit, the investigator or designee will review the patient and will undertake the necessary clinical and study procedures in the order outlined in the Schedule of Events. These include;

- Vital signs
- AE review
- Concomitant medication review
- Spirometry
- Collection of blood for laboratory analyses (to include full blood count, ESR, urea and electrolytes and liver function tests)
- Urinalysis

Once the results of the laboratory blood testing and urinalysis are available and have been reviewed the patient will be administered the study medication. In the case of treatment being contra-indicated (*i.e.* due to new infection or the development of new, unexplained haematuria) then the treatment visit can, at the discretion of the investigator, be postponed and rescheduled within the succeeding 2 weeks. If a greater than two week delay is required then the reason should be recorded in the CRF and the patient should proceed with the next planned visit. Wherever possible, even if treatment cannot be given, spirometry should be undertaken at the time of each planned visit.

## Treatment Visit (week 12)

Patients will be scheduled to receive their 4^th^ dose of Cyclophosphamide or placebo. The investigator or designee will review the patient and will undertake the necessary clinical and study procedures in the order outlined in the Schedule of Events. These include;

- Vital signs
- Concomitant medication review
- AE review
- Collection of blood for laboratory analyses (to include full blood count, ESR, urea and electrolytes and liver function tests)
- Blood for lymphocyte subsets
- Blood for Biomarker analysis
- Modified Rodnam’s Skin Score (scleroderma patients only)
- ECG
- Spirometry
- Lung Function (plethysmography and gas transfer)
- 6 Minute Walk Test
- Urinalysis

Once the results of the laboratory blood testing and urinalysis are available and have been reviewed the patient will be administered the study medication. In the case of treatment being contra-indicated (*i.e.* due to new infection or the development of new, unexplained haematuria) then the treatment visit can, at the discretion of the investigator, be postponed and rescheduled within the succeeding 2 weeks. If a greater than two week delay is required then the reason should be recorded in the CRF and the patient should proceed with the next planned visit. Wherever possible, even if treatment cannot be given, the study investigations should be undertaken at the time of the scheduled visit.

## Treatment Visit (weeks 16 and 20)

At each visit, the investigator or designee will review the patient and will undertake the necessary clinical and study procedures in the order outlined in the Schedule of Events. These include;

- Vital signs
- AE review
- Concomitant medication review
- Spirometry
- Collection of blood for laboratory analyses (to include full blood count, ESR, urea and electrolytes and liver function tests)
- Urinalysis

Once the results of the laboratory blood testing and urinalysis are available and have been reviewed the patient will be administered the study medication. In the case of treatment being contra-indicated (*i.e.* due to new infection or the development of new, unexplained haematuria) then the treatment visit can, at the discretion of the investigator, be postponed and rescheduled within the succeeding 2 weeks. If a greater than two week delay is required then the reason should be recorded in the CRF and the patient should proceed with the next planned visit. Wherever possible, even if treatment cannot be given, spirometry should be undertaken at the time of each planned visit.

## Primary End-Point Assessment (Week 24)

The primary endpoint assessment will be the first assessment following the end of the treatment phase of the study. The investigator or their designee will review the patient and will undertake the necessary clinical and study procedures in the order outlined in the Schedule of Events. These include;

- Vital signs
- Concomitant medication review
- AE review
- Collection of blood for laboratory analyses (to include full blood count, ESR, urea and electrolytes and liver function tests)
- Blood for lymphocyte subsets
- Blood for Biomarker analysis
- Global Disease activity score
- Quality of Life Questionnaires and Health Economic Patient Diary
- Modified Rodnam’s Skin Score (scleroderma patients only)
- ECG
- Spirometry
- Lung Function (plethysmography and gas transfer)
- 6 Minute Walk Test
- Urinalysis

Following this visit all subsequent treatment of patient’s Connective Tissue Disease (CTD) and associated ILD, including the use of additional immunosuppressant therapy, will be at the discretion of their usual treating physician.

## Final Follow-up Assessment (week 48)

Patients will be monitored for safety and efficacy through to Week 48 as per the Schedule of Events. The Final Follow up visit will occur ± 10 days unless otherwise noted and the following investigations will be undertaken;

- Vital signs
- Concomitant medication review
- AE review
- Collection of blood for laboratory analyses (to include full blood count, ESR, urea and electrolytes and liver function tests)
- Blood for lymphocyte subsets
- Blood for Biomarker analysis
- Global Disease activity score
- Quality of Life and Health Economics Questionnaires
- Modified Rodnam’s Skin Score (scleroderma patients only)
- ECG
- Spirometry
- Lung Function (plethysmography and gas transfer)
- 6 Minute Walk Test
- Urinalysis

## Unscheduled Visits

Patients may require an unscheduled visit in addition to the regular scheduled protocol visits (*e.g.* symptoms of infection, worsening of disease, or assessment of AEs). If a patient requires an unscheduled visit, the study centre will be strongly encouraged to undertake the following assessments:

- Physical examination
- Vital signs
- AE review
- Concomitant medication review
- Lung function (spirometry)
- Chest X-ray
- Collection of blood for laboratory analyses (to include full blood count, ESR, urea and electrolytes and liver function tests)
- Urinalysis
- ECG

**Table 2. Schedule of Events Table**

|  | **Screening**  **Visit(s)** | **R**  **A**  **N**  **D**  **O**  **M**  **I**  **S**  **E** | **Treatment Visits** | | | | | | | **Follow Up Visit** | |
| --- | --- | --- | --- | --- | --- | --- | --- | --- | --- | --- | --- |
| **TIME** | **1 - 2** |  | **Visit Day 0** | **Visit**  **Week 2** | **Visit week 4** | **Visit**  **Week 8** | **Visit**  **Week 12** | **Visit**  **Week 16** | **Visit**  **Week 20** | **Visit Week 24** | **Visit week 48** |
| **Consent** | X |  |  |  |  |  |  |  |  |  |  |
| **Study drug** |  |  | X | X | X | x | x | x | x |  |  |
| **Adverse event checking** |  |  | X | X | X | x | x | x | x | x | x |
| **Physical exam** | x |  |  |  |  |  |  |  |  |  |  |
| **Vital signs**  **(pulse, BP)** | x |  | x | x | x | x | x | x | x | x | x |
| **Routine bloods tests** | x |  | x | x | x | x | x | x | x | x | x |
| **Spirometry** | X |  | x | x | x | x | x | x | x | x | x |
| **Blood sample for lymphocyte subsets / Ig level** |  |  | X |  |  |  |  |  |  | x | x |
| **CK (in myositis patients)** | x |  | x | x | x | x | x | x | x | x | x |
| **Ig Levels** | x |  | x |  |  |  | x |  |  | x | x |
| **ECG** | x |  | x |  |  |  | x |  |  | x | x |
| **Lung function tests** | x |  | x |  |  |  | x |  |  | x | x |
| **6 MWT*** | x |  | x |  |  |  | x |  |  | x | x |
| **Urinalysis** | x |  | x | x | x | x | x | x | x | x | x |
| **Pregnancy test** | X |  |  |  |  |  |  |  |  |  |  |
| **QoL questionnaires** |  |  | x |  |  |  |  |  |  | x | x |
| **Health economic diary** |  |  | x |  |  |  |  |  |  |  | x |
| **mRSS (scleroderma)** |  |  | x |  |  |  | x |  |  | x | x |
| **Hepatitis B and C serology** | X |  |  |  |  |  |  |  |  |  |  |
| **Blood sample biomarker(s)** |  |  | X |  |  |  | X |  |  | X | X |
| **Blood sample genetics** |  |  | X |  |  |  |  |  |  |  |  |
| **Concomitant medication** | x |  | x | x | x | x | x | x | x | x | x |

Abbreviations; Routine bloods - full blood count, renal and liver function tests, ESR, CRP; Ig levels - immunoglobulin levels, creatinine kinase; ECG – electrocardiogram; HE – Health Economics; mRSS = modified Rodnan Skin Score; 6MWT = 6 minute walk test

Grey shaded square= tests are performed as routine clinical care in patients receiving standard therapy of monthly intravenous cyclophosphamide (for 6 months).

Lung function tests = plethysmography and gas transfer DLCO_2_,

## Assessment of Efficacy

The primary efficacy measurement will be change in FVC. Centres will be asked to undertake clinical trial spirometry on a single specified spirometer within their clinical physiology department or Clinical Trials Unit. Spirometry will be undertaken by a named individual or individuals who have had training to the standard recommended by the Association for Respiratory Technology and Physiology (ARTP).

Usage of spirometers must meet the standards outlined in the American Thoracic Society (ATS)/European Respiratory Society (ERS) guidelines (P05-12782), including daily calibration of the spirometer, and regular calibration of the calibration pump. Spirometry will be conducted while the patient is in a seated position. The test will be done in triplicate and selection of the best result done according to the guidelines. Spirometric results will be filed in the local medical records and will be available for review as required.

For each patient, pulmonary function testing will always start at approximately the same time of the day (with ±60 minutes maximum difference, time will be recorded). On days of clinic visits (including the screening visit), patients must refrain from strenuous activity at least 12 hours prior to pulmonary function testing.

Smoking will be discouraged throughout the study day (clinic visit) and will not be permitted in the 30-minute period prior to spirometry. Patients should also avoid cold temperatures, environmental smoke, dust, or areas with strong odours (*e.g*., perfumes). Washout of bronchodilators therapies (beta agonist or anticholinergic drugs) should be undertaken before spirometry: 24 hours for long acting and 8 hours for short acting bronchodilators.

## Assessment of Safety

The checking for the occurrence of adverse events and clinical endpoints will begin from randomisation and will continue for the individual patient until they complete their follow up at 48 weeks. At each study visit the Investigator or designee will make an assessment of safety and will specifically review the clinical history and investigation findings with regard to the occurrence of Adverse or Serious Adverse Events (SAEs). Details of adverse and clinical events will be captured on the trial eCRF.

## Research Blood Samples

All biological samples for future research will be collected and handled according to a study specific procedure, stored anonymously and labelled using a unique study number to permit accurate linkage to clinical data. Samples will be initially processed and stored at study sites in accordance with the study specific procedure for handling RECITAL biological samples, to facilitate transfer to the Royal Brompton Hospital (RBH) Biological Research Unit (BRU) Royal Brompton Hospital (RBH), Sydney Street, London, SW3 6NP.

The exploratory analysis of potential serum biomarkers and blood transcriptomics may be undertaken at; the Royal Brompton Hospital (RBH), Imperial College, London, a commercial research organization (CRO) or in collaborating academic institutions. Analysis of these samples may be undertaken after completion of the study and following assessment of the primary study outcome.

The storage of samples and use in future unspecified research will be performed in accordance with the Human Tissue Act 2004, and the RBH policy for ‘the acquisition, storage and use of Human biological specimens for research’. Stored samples may be used to assess future biomarkers for the risk of developing lung fibrosis and the prognosis of this condition.

We will ensure that participants are aware of this and details will be included in the Patient Information Sheet (PIS) and the consent form.

## Routine Safety Blood Tests

Routine safety blood tests including full blood count, U&E’s and liver function tests will be undertaken in the clinical laboratories at local sites according to local policies and procedures. Copies of local reference ranges will be collected.

## Other Routine Laboratory Tests

Other laboratory test results undertaken in the clinical laboratories at local sites according to local policies and procedures. Copies of local reference ranges will be collected and the investigator must notify the Trial Manager and ICTU of any subsequent changes in individual patient normalised values.

## Quality of Life Assessment

Quality of life will be assessed by self-administered questionnaires (adapted to the language and culture of each country). These are completed at baseline and repeated at first follow up visit for primary endpoint at 24 weeks and final follow up visit at 48 weeks.

The instruments used will be the SF36 and EQ5D (generic QoL questionnaires), the St George’s Respiratory Questionnaire, Kings Brief ILD and the Scleroderma Health Assessment Questionnaire which is disease specific.

# Treatment

## Name and description of each IMP

1. Cyclophosphamide in 0.9% sodium chloride intravenous infusion.
2. Rituximab 1000mg in 0.9% sodium chloride intravenous infusion.
3. Placebo 0.9% sodium chloride intravenous infusion.

## Source of IMPs including placebo

Bath ASU will be responsible for the manufacturing the IMPs and placebo which will be compliant with GMP requirements. They will purchase commercial supply of Rituximab (Roche Registration Ltd), Cyclophosphamide (Baxter Healthcare Ltd) and the placebo (0.9% sodium chloride Fresenius Kabi PL 08828/0084)). Following manufacturing, the study IMPs will be transported to study sites fully reconstituted in preparation for administration.

## Labelling

All study medication will be labelled with a unique identifier for drug accountability and in accordance with Annex 13 requirements.

## Storage and Administration

The investigational products must be stored securely in a temperature controlled unit between 2 – 8° C within a pharmacy department. Temperature logs should be kept and must be made available for monitoring and audit purposes. Specific instructions for storage and administration will be provided in a study specific IMP handling manual which will be provided in the Investigator Site File (ISF) and Pharmacy File.

## Accountability procedures for the IMP(s)

The site hospital Pharmacy departments will be responsible for maintaining & updating the study Pharmacy IMP Accountability Log, filed in the hospital pharmacy file. A study prescription form will document the dispensing of IMP to the research nurse or doctor; this should be retained in the pharmacy file. The research nurse or doctor is responsible for completing an IMP Administration Record Form at the point of administration; the completed form will be filed in the Investigator Site File (ISF).

Any IMP dispensed to the research nurse or doctor that has not been administered, *e.g.* due to cancellation, should be returned to pharmacy for destruction. All unused IMP, including unused returns, should be destroyed by the site pharmacy in accordance with local pharmacy practice, once agreed by the Sponsor. Destruction of unused IMP must be documented on the IMP Destruction Form and filed in the hospital pharmacy file.

All used or partially used IMP must be discarded in accordance with local cytotoxic waste disposal procedures; no used IMP shall be returned to pharmacy. A record of discarded used IMP should be made on the IMP Administration Form and filed in the Investigator Site File (ISF).

## Route of Administration, Dosage and Treatment Period(s) of the IMPs

Cyclophosphamide will be administered by intravenous infusion at a dose of 600 mg/m2 body surface area. The dose will be repeated every 4 weeks for a total of 6 doses. If clinically required individual doses may be delayed by up to 10 days. If longer delay is required then the planned dose should be omitted and the next scheduled dose given.

Rituximab will be administered by intravenous infusion at a dose of 1000 mg. The dose will be repeated at 14 days. This second dose may be delayed by up to 10 days. If it is not given within this time it should be omitted.

Placebo infusions will be administered in order to maintain the blind and all patients will receive 7 infusions in total.

Refer to the IMP Handling Manual for more details on administration and dosage.

## Dosage Modifications

For Cyclophosphamide dosage modifications in obese subjects (those with body mass index > 30), refer to the IMP Handling Manual.

## Assessment of Compliance

IMPs are administered intravenously. Completion of each infusion will be documented using an IMP Administration Record Form for each patient by study staff at each site. If infusions are discontinued for any reason, then the volume of remaining study medication should be recorded in the source data and on the eCRF.

## Post-trial IMP Arrangements

In the case of both Rituximab and Cyclophosphamide, the regimen administered during the study represents a complete course of therapy and so no provision has been made for further provision of either drug post-trial. If, in the opinion of participant’s regular treating physician, further Rituximab or Cyclophosphamide therapy is indicated then this will be provided as part of standard NHS care. As such, responsibility for securing funding for additional treatment will lie with patients’ local physicians.

## Permitted Concomitant Medication

### Non-IMP (nIMP)

The following nIMPS will be used in this study and administered to both groups when they receive Cyclophosphamide / Rituximab / placebo. All nIMPS are open label and generic forms can be used:

1. Mesna will be administered to patients in both groups at day 0 and monthly until week 20. Mesna 200 mg will be given by intravenous infusion in 100mls 0.9% sodium chloride over 30 minutes immediately prior to Cyclophosphamide / Rituximab / placebo. Additionally Mesna 400mg will be administered orally at 2 hours and 400mg at 6 hours post Cyclophosphamide / Rituximab / placebo infusion.
2. Hydrocortisone 100mg by intravenous injection to be given 30 minutes prior to Cyclophosphamide / Rituximab / placebo at day 0 and day 14.
3. Chlorphenamine 10mg by intravenous injection to be given 30 minutes prior Cyclophosphamide / Rituximab / placebo at day 0 and day 14.
4. Paracetamol 1g to be given orally 30 minutes prior to Cyclophosphamide / Rituximab / placebo at day 0 and day 14.
5. Patients will be offered Ondansetron an anti-emetic as required prior and for up to three days following the Cyclophosphamide / Rituximab / placebo infusions.

### Corticosteroids

Corticosteroids are strongly recommended for patients with CTD, all changes in corticosteroids should be documented in the eCRF

- It is anticipated that for patients with scleroderma, corticosteroids should be maintained at a stable dose of Prednisolone ≤10 mg daily.
- It is anticipated that individuals with inflammatory myositis will require high dose oral corticosteroids at initiation of therapy but that this should be weaned to ≤ 20mg Prednisolone daily by treatment week 12.
- Patients with MCTD should be maintained on a stable dose of ≤ 20 mg Prednisolone daily following entry in to the study.
- All changes in corticosteroid dosage should be recorded in the eCRF.

### Immunosuppressants

- At week 24, following completion of the treatment phase of the study and after measurement of the primary end-point, patients will be permitted to commence additional immunosuppressant therapy according to the recommendations of their treating physician.
- All other disease specific, non-immunosuppressant, therapies will be permitted for the duration of the study. Patients may also receive N-acetyl cysteine up to 600 mg t.d.s.

## Prohibited Concomitant Medication

- Pre-existing immunosuppresion (including Azathioprine, Mycophenolate Mofetil, Methotrexate, Cyclosporine, Hydroxychloroquine) will be stopped at least 14 days prior to randomisation.
- Between weeks 0 – 24, patients will not be permitted to receive additional immunosuppression (including oral agents, intravenous immunoglobulins, Hydroxychloroquine or other monoclonal antibody therapies) other than corticosteroids.

# Patient withdrawal

## Discontinuation of IMP Due to Progression of Underlying Disease

The study drug will be discontinued if, in the opinion of the local investigator/caring physician, an individual participant’s disease has progressed despite receiving study therapy. The decision regarding progression will rest with the local physician, but indicators of disease progression will include; worsening symptoms, progression of radiological changes and reduction in FVC of > 10% or Dlco of > 15% from baseline or reduction in resting oxygen saturations from baseline. Individuals with progressive disease will be un-blinded from the study, and if felt appropriate by their caring physician, may be offered the alternative treatment regimen on an open-label basis (*i.e.* patients receiving Rituximab will be offered Cyclophosphamide and those receiving Cyclophosphamide will be considered for Rituximab). Such treatment would be outside the study and, in the case of Rituximab may require individual funding to be sought from the appropriate NHS commissioning body. Similarly, in the case of individuals discontinuing treatment because of Adverse Events (AEs) the option will be open to the local caring physician to initiate open label treatment with the alternate treatment regimen (subject to the caveats regarding drug funding).

Subjects discontinuing the study drug will be invited to continue with planned monitoring and end of study visits. Complete protocol required data should be collected for all individuals who are randomized in to the study whether or not they receive their assigned treatment or discontinue the study prematurely. Subjects discontinuing study treatment will be asked to return for the primary endpoint (week 24) and final (week 48) follow up visits. Apart from Treatment week 12 they will not be required to attend any further treatment phase visits once treatment is discontinued.

## Permanent Discontinuation of IMP

Permanent discontinuation of study medication should occur in the following circumstances

- Consent withdrawn
- Pregnancy
- New diagnosis of tuberculosis, infective hepatitis or HIV.

## Possible Temporary Discontinuation of IMP

In the following cases withdrawal of study drug is highly recommended.

- Episode of severe infection requiring prolonged antibiotic treatment (>14 days) or hospitalisation
- New diagnosis of bladder cancer
- New occurrence of neurological symptoms suggesting a diagnosis of PML

However in special circumstances and after review of the clinical data, consultation with the appropriate specialist (urologist, neurologist) and members of the multi-disciplinary team, an appropriate risk benefit assessment and consultation with the patient the Investigator may decide not to withdraw the subject. All discussions and decisions should be documented in the medical notes.

In each case as this is an intention to treat trial, patients will be invited to continue attending study visits to allow for full collection of study data.

## Withdrawal from Trial Procedures and Incomplete Follow-up

Patients are free to withdraw consent from trial procedures and visits at any time resulting in incomplete patient follow-up. In these cases, permission will be sought to retain study data collected up until the time of withdrawal. Investigators must ascertain the reasons for the withdrawal, including discontinuation of study drug, withdrawal from study investigations and/or follow up, withdrawal due to adverse events, failure to attend, non-compliance, withdrawal of consent or other reasons. The RECITAL Trial Manager must be notified of withdrawals within 5 working days, unless withdrawal is due to a SAE, in which case the investigator will follow SAE reporting procedures.

# Endpoints

## Primary Endpoint

- Absolute change in FVC (expressed in mL) at week 24

## Secondary Endpoints

- Change from baseline in diffusing capacity for carbon monoxide (DLco) at 24 weeks
- Change from baseline in health related quality of life scores (SGRQ, SF-36, K-BILD)
- Change from baseline in global disease activity score
- Change in 6 minute walk distance over 48 weeks
- Change in FVC and DLco at 48 weeks
- Further analyses on FVC
  - Absolute categorical change of %FVC at 24 and 48 weeks (decrease by > 5%, increase by >5% and change within <5%)
  - Absolute categorical change of %FVC at 24 and 48 weeks (decrease by > 10%, increase by >10% and change within <10%)
  - 48 week rate of change in FVC
- Disease related mortality (adjudicated by steering committee at close of study)
- Overall survival
- Progression free survival (composite endpoint of mortality, transplant, treatment failure or decline in FVC > 10% compared to baseline)
- Treatment failure (as determined by need for transplant or rescue therapy with either open label Cyclophosphamide or Rituximab at any point until 48 weeks).
- Total corticosteroid requirement over 48 weeks
- Change from baseline in SpO2 at 24 and 48 weeks
- Healthcare utilisation during study period (visits to primary care, unscheduled hospital visits, emergency admissions)
- Scleroderma specific endpoints (change in scleroderma HAQ, modified Rodnan Skin Score (mRSS).

## Safety and Tolerability Endpoints

- Vital signs
- Physical examination
- Weight
- Laboratory tests (FBC, U&Es, LFTs, urinalysis)
- Adverse and serious adverse events
- Discontinuation of Rituximab or Cyclophosphamide due to intolerance or side effects.

## Exploratory endpoints

- Change in lymphocyte subsets relative to outcome
- Change in plasma cytokine levels following therapy and in relationship to markers of disease activity (FVC, DLco, QoL, global disease scores)
- Change in candidate serum biomarkers of fibrosis (to include KL-6, MMP1, MMP-7, Sp-A and Sp-D) following therapy.
- Outcome in relation to underlying CTD

## Health economics

This study will provide reliable data about the efficacy and safety of Rituximab compared to standard therapy. As Rituximab is considerably more expensive, the issue of cost effectiveness and affordability will arise if we show that it is more effective than standard treatments. In a study of this size it may be difficult to perform standard cost effectiveness analyses (*e.g.* cost per QALY) but we can estimate cost effectiveness using surrogate clinical outcomes and also reliably estimate costs for care in the two treatment groups including costs of drug, tests and investigations, health care visits (hospital, GP, clinic). We will also have a range of patient based outcomes using validated questionnaires. Working with the health economic team at the University of East Anglia, the health economic analysis will deliver a high quality analysis using standard techniques to inform our understanding of the cost effectiveness of the new treatment.

# pharmacovigilAnce

## Pharmacovigilance and Safety Reporting

The study Sponsor is responsible for pharmacovigilance and will provide all sites with the Sponsor’s pharmocovigilance Standard Operating Procedure (SOP) for recording and reporting of Adverse Events (AEs). It is the responsibility of the Sponsor to ensure that all Serious Adverse Events (SAEs) are reported to Chief Investigator (CI) (or designated deputy) for review. The Sponsor is also responsible for expedited reporting of Suspected Unexpected Serious Adverse Reactions (SUSARs) in accordance with statutory regulations. The Chief Investigator’s designated deputies will be selected investigators from the trial sites, who can provide clinical expertise to adjudicate Serious Adverse Events (SAEs) for seriousness, causality and relatedness, and advise whether expedited reporting is required. Additional pharmacovigilance guidance will be provided in accompanying documents, including eCRFs.

## Definitions

### Adverse Event (AE) Definition

Any untoward medical occurrence in a patient or clinical trial patient who is administered an IMP and which does not necessarily have a causal relationship with this treatment. (*i.e.* any unfavorable or unintended change in the structure (signs), function (symptoms), or chemistry (lab data) in a patient to whom an IMP has been administered, including occurrences unrelated to that product)

## Adverse Reaction (AR) Definition

Any untoward and unintended responses to an IMP related to any dose administered. (*i.e.* any unfavourable or unintended change in the structure (signs), function (symptoms), or chemistry (lab data) in a patient to whom an IMP has been administered and related to any dose administered).

## Serious Adverse Event (SAE) or Serious Adverse Reaction (SAR)

Any Adverse Event or Reaction in a trial patient that:

- Results in death; or
- Is life-threatening (places the patient, in the view of the Investigator, at immediate risk of death)
- Requires hospitalisation or prolongation of existing hospitalisation (hospitalisation is defined as an inpatient admission, regardless of length of stay; even if it is a precautionary measure for observation; including hospitalisation for an elective procedure, for a pre-existing condition)
- Results in persistent or significant disability or incapacity (substantial disruption of one’s ability to conduct normal life functions)
- Consists of a congenital anomaly or birth defect (in offspring of patients or their parents taking the IMP regardless of time of diagnosis).

*Comments from ENTR/CT 3Annex 1:*

*Life-threatening in the definition of a serious adverse event or serious adverse reaction refers to an event in which the subject was at risk of death at the time of event; it does not refer to an event which hypothetically might have caused death if it were more severe.*

*Important medical events that may not be immediately life-threatening or result in death or hospitalisation but may jeopardise the patient or may require intervention to prevent one of the outcomes listed in the definition of serious will also be considered serious.*

## Suspected Unexpected Serious Adverse Reaction (SUSAR)

An Adverse Reaction which is classed in nature as both serious and unexpected. An Unexpected Adverse Reaction is an Adverse Reaction, when both the nature and severity of the event is not consistent with the information about the medicinal product *i.e.* the information available in the Summary of Product Characteristics (SMPC) for Rituximab, Cyclophosphamide, sodium chloride.

## Expedited Reporting of SUSARs

Investigators are required to notify all SUSARs immediately to the Trial Manager within 24hrs of becoming aware of the event. Investigators must complete the appropriate SAE form on the eCRF and an automatic email notification will be sent to the Trial Manager, CI and Sponsor. The Trial Manager will ensure the SUSAR report is un-blinded and is reviewed by the CI or designee within 2 days and adjudicate whether the event constitutes a SUSAR.

The Trial Manager will ensure that fatal or life threatening SUSARs are reported to the MHRA and the main REC as soon as possible, but no later than 7 calendar days after the receipt of the eSAE report. Any additional information will be reported within 8 days of sending the first report. The Trial Manager must report all other SUSARs and safety issues to the MHRA and main REC, as soon as possible but no later than 15 calendar days after the Sponsor has first knowledge of the minimum criteria for expediting reporting.

## Recording and Reporting of Adverse Events (AEs) – (including SAE/Rs)

1. There is routine monitoring of patient status, blood tests (FBC, U&Es, LFTs) and lung function and data is captured on the eCRF.
2. All SAEs/SARs (including expected) will be reported on the eCRF by the investigator / or designee within 24 hours of their becoming aware of the event. The eCRF will send a notification of each SAE report to the Sponsor and the CI (or designee) who will review the report within 2 working days of receipt.
3. All SAEs, whether related or unrelated to the treatment will be recorded in the hospital notes and eCRF. The Sponsor will have access to SAEs *via* the eCRF.
4. If the Investigator suspects that the disease has progressed faster due to the administration of the IMP, then s/he will report this as an unexpected AE.

## Expected SAE/Rs

### Expected SAE/Rs Related to IMP

Expected Adverse Reactions are known side effects of the IMP reported in SmPCs of Cyclophosphamide, Rituximab and sodium chloride.

### Expected SAEs Related to Underlying Disease

Events that are expected and related to underlying disease include study endpoints and disease progression or worsening of pre-existing respiratory or rheumatological symptoms.

## Severity of Adverse Events

**Severity** will be described using the following categories:

| **Mild** | The adverse event does not interfere with the patient’s daily routine, and does not require intervention; it causes slight discomfort. |
| --- | --- |
| **Moderate** | The adverse event interferes with some aspects of the patient’s routine, or requires intervention, but is not damaging to health; it causes moderate discomfort. |
| **Severe** | The adverse event results in alteration, discomfort or disability which is clearly damaging to health. |

## 12.10 Causality / Relationship to Treatment

The assessment of relationship of AEs to the administration of IMP is a clinical decision based on all available information at the time of the completion of the eCRF. The following categories will be used:

| **Definitely** | There is clear evidence to suggest a causal relationship, and other possible contributing factors can be ruled out. |
| --- | --- |
| **Probably** | There is evidence to suggest a causal relationship, and the influence of other factors is unlikely. |
| **Possibly** | There is some evidence to suggest a causal relationship (*e.g.* the event occurred within a reasonable time after administration of the trial medication). However, the influence of other factors may have contributed to the event (*i.e.* the patient’s clinical condition, other concomitant events). |
| **Unlikely** | There is little evidence to suggest there is a causal relationship (*e.g.* the event did not occur within a reasonable time after administration of the trial medication). There is another reasonable explanation for the event (*e.g.* the patient’s clinical condition, other concomitant treatments). |
| **Not related** | There is no evidence of any causal relationship. |

## 12.11 The type and duration of the follow-up of patients after AEs

Follow up and care for patients suffering an Adverse Drug Reaction (ADR) will be provided as required by clinical need. All SAEs will be followed up every two weeks until resolution.

### 12.12 Development Safety Update Reports (DSURs)

The Sponsor will prepare the DSUR in collaboration with the Chief Investigator (CI) in accordance with regulatory requirements. It will be reviewed by the Clinical Research Oversight Committee (Clin-ROC).

### 12.13 Annual Progress Reports (APRs)

The Sponsor will prepare the APR in collaboration with the CI. It will be reviewed by the Sponsor and sent to the main REC within 30 days of the anniversary date on which the favorable opinion was given by the Ethics Committee, and annually until the trial is declared ended.

## 12.14 Pregnancy reporting

Rituximab and Cyclophosphamide are contra-indicated in pregnancy and for this reason all female patients of child bearing age will be asked to undertake a pregnancy test at the screening study visit. In the event of a pregnancy during the study this will be recorded in the source data file and will be reported within 72 hours to the Sponsor, using the Pregnancy Reporting Form in the eCRF. Patients will be excluded from further administration of IMP but will be invited to attend future study visits. Outcome of the pregnancy will be followed up by trial staff.

## 12.15 Safety Reporting Requirements for nIMPS

There are a number of rescue nIMPS that are administered prior to administration of IMP (Cyclophosphamide/Rituximab/sodium chloride) as detailed in section 9.10.1. The following reporting arrangements for nIMPS apply:

1. SUSARS related to nIMPs where there is a possibility of an interaction between a nIMP and an IMP must be reported as SUSARS (there is therefore no need to report a SUSAR related to a nIMP if there is no suspected interaction between the nIMP and IMP).
2. If a SUSAR occurs, and it might be linked to either a nIMP or an IMP, but cannot be attributed to only one of these, the SUSAR must be reported,
3. If an adverse reaction associated with a nIMP is likely to affect the safety of the trial subjects, then an urgent safety reporting measure must be undertaken section .
4. All other nIMP suspected Adverse Drug Reactions (ADR) or side effects will be reported by PIs through the MHRA yellow card system.

## 12.16 Reporting Urgent Safety Measures

The Investigator may take appropriate urgent safety measures in order to protect the patients of a clinical trial against any immediate hazard to their health or safety. If measures are taken, the Investigator should contact the Sponsor immediately and within 24 hours. The Sponsor will review the “urgent safety measure” with the CI and ensure an appropriate report is made to the MHRA and REC within the specified timeline of 3 days from when the safety measure was implemented. Investigators will notify the Sponsor of all urgent safety measures and reports.

## 12.17 Notification of Serious Breaches of GCP

The Investigator is responsible for notifying the Sponsor of any serious breaches of GCP. The Sponsor will review any serious breaches of GCP with the CI and notify the MHRA and REC in accordance with the specified timelines. Investigators will notify the Sponsor of all serious breaches of GCP.

# Data Management

## Data Collection

Data will be collected on an electronic Case Report Form (eCRF) system. The InForm system will be used to develop the eCRF and will be designed in accordance with the requirements of the clinical trial protocol and will comply with regulatory requirements. Local personnel will be trained on the InForm system. Access will be restricted to site personnel, trial managers, trial monitors and the data management team. Personnel will have individual logon and passwords. It is the Investigator’s responsibility to ensure the accuracy of all data entered and recorded in the eCRFs. Trial monitors will check the accuracy of the eCRF data against source documents

## Source Data

It is anticipated that the majority of source data (medical progress notes and letters, tests and investigations) will be filed in the individual patients medical records. Any deviation from source data being present in the medical notes will be identified and documented. The eCRF and source documents must be available at all times for review by the Sponsor’s clinical trial monitor, auditors and for inspection by the Medicines Health Regulatory Agency. The accuracy of eCRF data will be verified by review of the source documents and details will be provided in the trial Monitoring Report.

## Archiving

Clinical trial documents will be archived and held by the Sponsor for twenty five years after study closure in accordance with the Standard Operating Procedures (SOP) of the Royal Brompton and Harefield NHS Foundation Trust and in compliance with the principles of GCP.

In compliance with the ICH/GCP guidelines, regulations and in accordance with the Royal Brompton SOP, the Chief or local Principal Investigator will maintain all records and documents regarding the conduct of the study. These will be retained for at least 5 years or for longer if required. If the responsible investigator is no longer able to maintain the study records, a second person will be nominated to take over this responsibility.

The Trial Master File (TMF) and trial documents held by the Sponsor on behalf of the CI shall be archived at secure off-site archiving facility sub-contracted by the Royal Brompton Hospital (RBH). This archive shall include all trial databases and associated encryption codes.

# Statistical issues

## Sample Size

Previous studies of intravenous Cyclophosphamide in SSc demonstrated a 1% decline in FVC at twelve months, with a coefficient of variation of 7.8%. Our observational data and a previous non-randomised study of Rituximab (used as rescue therapy in those failing treatment with Cyclophosphamide) suggest improvements in FVC at 6 - 12 months of between 9.5 - 20% compared to baseline[^19^](#_ENREF_19). Using 1:1 randomisation, a sample size of 52 patients in each group will have a 90% power to detect a 5% difference (approximately 140 mls) between groups at 24 weeks in the change in FVC (as measured in mL) with a significance level (alpha) of 0.05 (two tailed). Anticipating a drop-out rate of 10% our target recruitment is therefore 58 patients in each arm of the study. On the basis of data derived in other interstitial lung disease 5% change in FVC is associated with change in long term prognosis and can therefore be considered a clinically meaningful difference between the two groups[^26^](#_ENREF_26). Given the number of individuals treated with Cyclophosphamide at our unit and in units that will be participating in the study, this number is feasible to deliver within the planned trial timelines.

## Statistical Analysis Plan

This is a phase 2, multicentre, randomized, double-blind, double-dummy comparison of Rituximab against Cyclophosphamide in the treatment of CTD associated ILD. The study is designed to test the hypothesis that Rituximab is superior to Cyclophosphamide in the treatment of CTD associated ILD in improving lung function as measured by FVC. The primary end-point is the absolute change in FVC (measured in mL) at week 24. Full details of the trial data analysis will be provided in the Statistical Analysis Plan.

## Primary Outcome

Analysis of the primary outcome will be by intention to treat. Analysis of covariance (ANCOVA), including baseline FVC, the stratification factors for randomization and treatment group will be used as independent variables for the analysis. The hypothesis to be tested is that Rituximab is superior to Cyclophosphamide. The study will be considered positive if statistical significance at the level of 0.05 (two tailed) is achieved.

The effect of missing data on the primary endpoint will be investigated using pattern-mixture models. Depending on the amount of missing data a multiple-imputation methods may be considered.

## Secondary Outcomes

Key secondary end-points include change in diffusing capacity for carbon monoxide (DLco), SGRQ and 6MWD from baseline at week 48, mortality, progression free survival and healthcare utilization. Change in continuous physiological variables between baseline and 48 weeks will be assessed by ANCOVA with the same independent variables used for the primary analysis. Categorical change in physiological variables (e.g. <10% improvement, >10% improvement) will be measured using chi-squared tests. Mortality and progression free survival will be measured using Kaplan-Meier estimates. Log rank test will be used to compare treatment groups and Cox model will be used to determine hazard ratios for survival analyses.

Exploratory biomarker analyses will use linear and logistic regression as appropriate. At a minimum all data will be listed and summarized. An analysis of outcome (as measured by change in FVC) according to underlying CTD will be undertaken.

## Safety Analysis

All treated patients will be included in the safety analysis. In general safety analyses will be descriptive and no hypothesis testing is planned. Data will be analysed at regular, pre-specified intervals by the DMC.

Time to trial drug discontinuation will be analyzed using Kaplan-Meier estimates. Frequency, severity and causal relationship of adverse events will be tabulated by system organ class (SOC) and preferred term (PT) after coding according to the current version of the Medical Dictionary for Drug Regulatory Activities (MedDRA). Statistical analysis and reporting of adverse events will concentrate on treatment emergent adverse events. To this end, all adverse events with an onset after the first dose of study medication up to a period of 28 days (inclusive) after the last dose of study medication will be considered ‘treatment emergent’ and will be assigned to the treatment phase for evaluation. Adverse events that start before first drug intake and deteriorate under treatment will also be considered as ‘treatment-emergent’. Other adverse events will be assigned either to the screening or post treatment, or post study phase, as appropriate.

For laboratory evaluations, treatment groups will be compared descriptively with regard to the distribution of the parameters as well as to frequency and percentage of patients with abnormal values (outside the reference range) or clinically relevant abnormal values. Changes from baseline in vital signs parameters as well as weight will be summarized by treatment group.

## Interim analyses

No formal interim analysis is planned. A regular review of safety data will be conducted to monitor the safety of patients in the trial. A Data Monitoring Committee (DMC) will follow number of deaths, early discontinuation due to Adverse Events (AEs) and Serious Adverse Events (SAEs) in an un-blinded fashion. The first meeting will be held to review all available data after the 12^th^ randomized patient has completed the week 24 visit and periodically thereafter. The complete details will be outlined in a DMC charter to be agreed by the DMC members at the start of the study.

# Trial Management and committees

## Trial Management and Structure

The study sponsor is The Royal Brompton & Harefield NHS Foundation Trust (RB&HFT) who has overseen the design of the study and will have oversight of the trial. The Chief Investigator is Dr Toby Maher at the Royal Brompton Hospital (RBH). A Trial Steering Committee (TSC) and Data Monitoring Committee (DMC), and an Operational Trial Management Group (TMG) will be convened to oversee the trial. Trial management will be provided by the study Sponsor and under the overarching oversight of the Imperial College Trials Unit (ICTU).

## Trial Steering Committee (TSC)

An independent Trial Steering Committee (TSC) will be established to oversee the conduct of the study. It is anticipated that the TSC will comprise the lead investigators, an independent chair, two additional independent members, at least one of whom will be a patient/public representative, ICTU representative. The TSC will develop terms of reference outlining their responsibilities and operational details. The TSC will meet before the trial commences and as required during the course of the study. Already recruited TSC members have participated in clinical trial design and have provided data to guide feasibility and safety.

## Data Monitoring Committee (DMC)

An independent Data Monitoring Committee (DMC) will be established to review safety data during the course of the study. The DMC will develop a charter outlining their responsibilities and operational details. The first DMC meeting will be held early in the trial and thereafter as required.

The Investigator(s)/institution(s) will permit trial-related monitoring, audits, REC review, and regulatory inspection(s), providing direct access to source data/documents. Trial participants are informed of this during the informed consent discussion. Participants will consent to provide access to their medical notes

## Trial Management

The study will be managed jointly by ICTU and the study Sponsor; the Sponsor will appoint a dedicated trial manager who will be based at RBHT but operationally accountable to the ICTU senior management team and conduct the study according to ICTU SOPs. The trial manager will assist the CI in preparing the final protocol, submitting the Ethics, MHRA and R&D applications, preparing the investigator, pharmacy and other study specific manuals. The manager will ensure that the trial runs according to the pre-agreed timetable, recruitment targets are met, the Case Report Forms (CRFs) are completed accurately, compliance with relevant ethical and other regulatory standards, and that all aspects of the study are performed to the highest quality. The trial manager will also assist in the training of investigators at the start-up of the study and in performing monitoring during the study. The manager will be a contact point to provide support and guidance to the participating centres throughout the study.

## Investigators’ Responsibilities

Investigators must ensure that local Institutional approval has been obtained as well as Agreements signed off by their Institution prior to the start of the study. Investigators are responsible for performing the study in accordance with the European Clinical Trials Directive, all local laws and guidelines. Investigators are required to ensure compliance to the Clinical Trial Protocol, CRFs, Investigators File and any other study instructions as required by the Sponsor or its representatives. Investigators are required to ensure the accuracy of the trial data according to the instructions provided investigators are required to allow access to study documentation or source data on request for monitoring visits and audits performed by the ICTU, the sponsor or any regulatory authorities. The investigator may appoint co-investigators to assist with conduct of the study locally. All co-investigators must be listed as members of the research team and appropriately trained. The investigator has overall responsibility for ensuring the conduct of the study locally.

## Patient and Public Involvement

The involvement of service users has been an important driver for the development of this study. The Raynaud’s and Scleroderma Society have been involved in protocol development and the society’s Chief Executive, Mrs Anne Mawdsley, will be a steering committee member. This involvement had focused on the suitability of the research question, the potential benefits to CTD-ILD sufferers and the practical elements of study deign including the recruitment targets, visit schedule and the planned double dummy administration. A second service user/carer will be sought to ensure that patient needs are fully addressed throughout the trial set up and management (*i.e.* if one member cannot attend the TSC). Part of this set up will involve helping to further develop the final protocol and to develop Patient Information Sheet (PIS) and the ethics application. As a service user, Mrs Mawdsley has advised on the level of involvement that would be appropriate during the trial and this has been taken into account in the study design, conduct and implementation.

# regulatory, Ethical and legal issues

## Study Conduct

The study conduct shall comply with all relevant laws of the EU and all relevant laws and statutes of the UK including but not limited to, the Human Rights Act 1998, the Data Protection Act 1998, the Medicines Act 1968, the Medicines for Human Use (Clinical Trial) Regulations 2004, and with all relevant guidance relating to medicines and clinical studies from time to time in force including, but not limited to, the ICH GCP, the World Medical Association Declaration of Helsinki entitled 'Ethical Principles for Medical Research Involving Human Patients' (2008 Version), the NHS Research Governance Framework for Health and Social Care (Version 2, April 2005).

## Regulatory Requirements

This study is a randomised trial of an Investigational Medicinal Product (IMP) and as such will need to comply with the European Clinical Trials Directive and the Medicines for Human Use (Clinical Trials) Regulations 2004 and its subsequent amendments. An application for Clinical Trials Authorisation (CTA) will be submitted to the Medicines and Healthcare Regulatory Agency (MHRA) prior to starting the study. The study will be registered on a clinical trial database prior to the start of the study. Protocol amendments must be approved by the regulatory authority. SUSAR reports, annual safety updates and notification of the end of the study will be sent to the regulatory authorities.

## Ethical Requirements

The study protocol, patient information sheet (PIS) and informed consent forms (ICF) will be submitted to the UK National Research Ethics Committees for approval. Protocol amendments must be approved by the Research Ethics Committee before they can be implemented. Changes to the PIS and or ICF must be approved by the Research Ethics Committee (REC). No deviation from the protocol will be implemented without the prior review and approval of the REC, except where it may be necessary to eliminate an immediate hazard to a research patient. In such case, the deviation will be reported to the REC as soon as possible. Annual reports and notification of the end of the study will be sent to the Research Ethics Committee in accordance with their guidelines.

## Institutional Approval

NHS R&D site specific approval will be required and a Clinical Trial Agreement between the Sponsor and each of the participating sites is required before the study can commence at the individual site.

## Funding

This study is funded by the Efficacy and Mechanism Evaluation Programme of the National Institute for Health Research (EME (NIHR)).

Participants will not be paid to participate in the trial. Travel expenses will only be offered for hospital visits in excess of usual care.

## Insurance and Indemnity

Insurance and indemnity for study participants and study staff is covered within the NHS Indemnity Arrangements for clinical negligence claims in the NHS, issued under cover of HSG (96)48. There are no special compensation arrangements, but study participants may have recourse through the NHS complaints procedures. NHS bodies are liable for clinical negligence and other negligent harm to individuals covered by their duty of care. NHS Institutions employing researchers are liable for negligent harm caused by the design of studies they initiate. The provision of such indemnity for negligent harm should be stated to the participant.

## Confidentiality and Data Protection

The eCRFs will not bear the patient’s name or other personal identifiable data. The patient’s initials, Date of Birth (DOB) and trial Identification Number (ID), will be used for identification.

All study staff and investigators will endeavour to protect the patient’s rights to privacy and will adhere to the Data Protection Act, 1998. Only the minimum required information for the purposes of the study shall be collected. Documents will be held securely in a locked room. Investigators shall permit access to patient’s source documents and other study documentation for the purposes of monitoring, auditing or inspection by the Trial Management group, the Sponsor, authorised representatives of the Sponsor or Regulatory Authorities.

Computer held data including the study database will be held securely and password protected. All data will be stored on a secure dedicated web server. Access will be restricted by user identifiers and passwords. Information about the study in the participant’s medical records / hospital notes will be treated confidentially in the same way as all other confidential medical information.

Individual participant’s medical information obtained as a result of this study will be considered confidential and disclosure to third parties is prohibited with the exceptions noted above. Participant confidentiality will be further ensured by utilising identification code numbers to correspond to treatment data in the computer files. Such medical information may be given to the participant’s medical team and all appropriate medical personnel responsible for the participant’s welfare.

# Quality Control and assurance

The quality control and assurance will be according to ICTU Standard Operating Procedures.

## Monitoring

The trial will be monitored according to the monitoring plan which will be developed in accordance with ICTU SOPs and based on the trial risk assessment. Monitoring will include but is not limited to, checks on consent forms, Source Data Verification (SDV), investigator site file and pharmacy file, local procedures, delegation logs, IMP storage and accountability, IMP destruction.

## Audits and Inspections

For the purpose of ensuring compliance with the Clinical Trial Protocol, Good Clinical Practice (GCP) and applicable regulatory requirements, the investigator should permit auditing by or on the behalf of the sponsor and inspection by applicable regulatory authorities. The investigator agrees to allow the auditors/inspectors to have direct access to his/her study records for review, it being understood that this person(s) are bound by professional confidentiality, and as such will not disclose any personal identity or personal medical information. The investigator will make every effort to help with the performance of the audits and inspections, giving access to all necessary facilities, data and documents. As soon as the investigator is notified of a future inspection by the authorities, he will inform the sponsor and authorise the sponsor to participate in this inspection. The confidentiality of the data verified and the protection of the patients should be respected during these inspections. The sponsor or its representative will immediately communicate any results and information arising from the inspections by the regulatory authorities. The investigator shall take appropriate measures required by the sponsor to take correctives actions for all problems found during the audit or inspections.

# End of trial

## Planned Termination

The trial will end when the last patient randomised has completed their last visit at 48 weeks (LPLV).

## Premature Termination by Sponsor

The trial may be terminated prematurely:

- If the recruitment target cannot be met within the projected recruitment phase

- If the TSC believe the trial is no longer clinically relevant

- If there are significant concerns regarding the benefit / risk to the patient is in doubt.

The trial can be terminated by the sponsor or its representative at an individual Trial Site(s) if:

- the Trial Site(s) cannot comply with the requirements of the protocol,

- the Trial Site(s) is unable to comply with the required data standards,

- the required recruitment rate is not met.

If (after discussion between the TSC, the sponsor and ICTU) the trial is terminated in a trial site(s) because of repeated serious protocol violations, or gross violations of data standards, all patients entered in the trial site(s) concerned will be excluded from analysis.

## Premature Termination by the Investigator

The investigator must provide prior notice to the sponsor (or its representative) of his/her decision and give the reason in writing. In all cases the appropriate Ethics Committee and appropriate regulatory authority should be informed.

# Data Ownership and Publication Policy

Data ownership rights will lie with the sponsor. The results of this study will be drafted as a series of scientific papers. In addition results will be presented at local and international conferences and seminars. The Trial Steering Committee (TSC) will be responsible for approval of all scientific papers arising from the study prior to submission for publication. All publications and presentations will make appropriate acknowledgement of the contribution of the collaborative group. At the end of the study, patients will be able to request a copy of the results of the study from the investigator at that site. Individuals who participated in the study will not be identifiable from any of these outputs.

# References

1. Tyndall AJ, Bannert B, Vonk M, et al. Causes and risk factors for death in systemic sclerosis: a study from the EULAR Scleroderma Trials and Research (EUSTAR) database. Ann Rheum Dis 2010;69:1809-15.
2. Luzina IG, Atamas SP, Wise R, Wigley FM, Xiao HQ, White B. Gene expression in bronchoalveolar lavage cells from scleroderma patients. Am J Respir Cell Mol Biol 2002;26:549-57.
3. Lafyatis R, O'Hara C, Feghali-Bostwick CA, Matteson E. B cell infiltration in systemic sclerosis-associated interstitial lung disease. Arthritis and rheumatism 2007;56:3167-8.
4. Bosello S, De Santis M, Lama G, et al. B cell depletion in diffuse progressive systemic sclerosis: safety, skin score modification and IL-6 modulation in an up to thirty-six months follow-up open-label trial. Arthritis Res Ther 2010;12:R54.
5. Hoyles RK, Ellis RW, Wellsbury J, et al. A multicenter, prospective, randomized, double-blind, placebo-controlled trial of corticosteroids and intravenous cyclophosphamide followed by oral azathioprine for the treatment of pulmonary fibrosis in scleroderma. Arthritis and rheumatism 2006;54:3962-70.
6. Tashkin DP, Elashoff R, Clements PJ, et al. Cyclophosphamide versus placebo in scleroderma lung disease. N Engl J Med 2006;354:2655-66.
7. Perosa F, Prete M, Racanelli V, Dammacco F. CD20-depleting therapy in autoimmune diseases: from basic research to the clinic. J Intern Med 2010;267:260-77.
8. Leandro MJ, Cambridge G, Ehrenstein MR, Edwards JC. Reconstitution of peripheral blood B cells after depletion with rituximab in patients with rheumatoid arthritis. Arthritis and rheumatism 2006;54:613-20.
9. Edwards JC, Leandro MJ, Cambridge G. B lymphocyte depletion therapy with rituximab in rheumatoid arthritis. Rheum Dis Clin North Am 2004;30:393-403, viii.
10. Emery P, Fleischmann R, Filipowicz-Sosnowska A, et al. The efficacy and safety of rituximab in patients with active rheumatoid arthritis despite methotrexate treatment: results of a phase IIB randomized, double-blind, placebo-controlled, dose-ranging trial. Arthritis and rheumatism 2006;54:1390-400.
11. Cohen SB, Emery P, Greenwald MW, et al. Rituximab for rheumatoid arthritis refractory to anti-tumor necrosis factor therapy: Results of a multicenter, randomized, double-blind, placebo-controlled, phase III trial evaluating primary efficacy and safety at twenty-four weeks. Arthritis and rheumatism 2006;54:2793-806.
12. Jones RB, Tervaert JW, Hauser T, et al. Rituximab versus cyclophosphamide in ANCA-associated renal vasculitis. N Engl J Med 2010;363:211-20.
13. Stone JH, Merkel PA, Spiera R, et al. Rituximab versus cyclophosphamide for ANCA-associated vasculitis. N Engl J Med 2010;363:221-32.
14. Arnold DM, Dentali F, Crowther MA, et al. Systematic review: efficacy and safety of rituximab for adults with idiopathic thrombocytopenic purpura. Ann Intern Med 2007;146:25-33.
15. Sem M, Molberg O, Lund MB, Gran JT. Rituximab treatment of the anti-synthetase syndrome: a retrospective case series. Rheumatology (Oxford) 2009;48:968-71.
16. Daoussis D, Liossis SN, Tsamandas AC, et al. Is there a role for B-cell depletion as therapy for scleroderma? A case report and review of the literature. Semin Arthritis Rheum 2010;40:127-36.
17. Daoussis D, Liossis SN, Tsamandas AC, et al. Experience with rituximab in scleroderma: results from a 1-year, proof-of-principle study. Rheumatology (Oxford) 2010;49:271-80.
18. Keir GJ, Maher TM, Hansell DM, et al. Severe interstitial lung disease in connective tissue disease: Rituximab as rescue therapy. Eur Respir J 2012.
19. Goh NS, Desai SR, Veeraraghavan S, et al. Interstitial lung disease in systemic sclerosis: a simple staging system. American journal of respiratory and critical care medicine 2008;177:1248-54.
20. Beirne P, Pantelidis P, Charles P, et al. Multiplex immune serum biomarker profiling in sarcoidosis and systemic sclerosis. Eur Respir J 2009;34:1376-82.
21. LeRoy EC, Black C, Fleischmajer R, et al. Scleroderma (systemic sclerosis): classification, subsets and pathogenesis. The Journal of rheumatology 1988;15:202-5.
22. Bohan A, Peter JB. Polymyositis and dermatomyositis (second of two parts). N Engl J Med 1975;292:403-7.
23. Bohan A, Peter JB. Polymyositis and dermatomyositis (first of two parts). N Engl J Med 1975;292:344-7.
24. Alarcon-Segovia D, Cardiel MH. Comparison between 3 diagnostic criteria for mixed connective tissue disease. Study of 593 patients. The Journal of rheumatology 1989;16:328-34.
25. Zappala CJ, Latsi PI, Nicholson AG, et al. Marginal decline in forced vital capacity is associated with a poor outcome in idiopathic pulmonary fibrosis. Eur Respir J 2010;35:830-6.

# Appendix 1: Specific Adverse Events

Routine monitoring will be performed on patients including vital status, blood tests (FBC, U&Es, LFTs), spirometry. The blood tests detailed below are of specific safety interest and regular checks will be performed to identify values outside the cut off value and will be reviewed by the CI and DMC.

| **Description** | **Cut off value** |
| --- | --- |
| White cell count | <4 x10^9^/L |
| Neutrophils | 1.5 x10^9^/L |
| Creatinine | > ULN or 1.5Xbaseline value |
| Alanine aminotransferase (ALT) | > 2XULN |
| Alkaline phosphatase (ALP) | > 2XULN |
| Bilirubin | > 2XULN |
