# Supplementary material for: Rituximab versus cyclophosphamide for the treatment of connective tissue disease-associated interstitial lung disease (RECITAL): study protocol for a randomised controlled trial
Source: Trials. 2017 Jun 15;18:275. doi: 10.1186/s13063-017-2016-2 (PMC5471887; doi:10.1186/s13063-017-2016-2)
Supplement: Supplementary file 3 — Patient Information Sheet. Version 6.0 15.10.2014. (DOC 217 kb) [file 13063_2017_2016_MOESM3_ESM.doc]

*(insert on local Trust headed paper)*

**PATIENT INFORMATION SHEET**

**Part 1**

**Title of Project:** A randomized, double blind controlled trial comparing Rituximab against intravenous Cyclophosphamide in connective tissue disease associated interstitial lung disease

**Short Title:** The RECITAL Study

**Invitation paragraph**

We would like to invite you to take part in a research study. Before you make your decision it is important for you to understand why this research is being done and what it will involve. One of our team will go through the information sheet with you and answer any questions you have. Please take as much time as you need to read the following information carefully and discuss it with others if you wish. Part 1 tells you the purpose of this study and what will happen to you if you take part. Part 2 gives you more detailed information about the conduct of the study.

This study will involve intravenous drugs either in addition to or in place of the medication you are currently taking (this will have been discussed with you by your consultant). As the next step in your treatment regimen recommended by your doctor would be to commence cyclophosphamide, we would like to invite you to consider participating in this study. If you participate you will have a 50:50 chance of receiving this standard treatment versus the newer treatment being studied in this trial.

This is your copy of the information sheet to keep for future reference. If you decide to take part you will also be given a copy of the consent form that you signed.

Thank you for taking the time to read this information sheet.

**What is the purpose of the study?**

We are undertaking a research study to determine the best drug (cyclophosphamide or rituximab) with which to treat connective tissue disease associated interstitial lung disease (CTD-ILD). This study will involve about 116 patients in several hospitals in England.

In connective tissue disease (CTD) (including diseases such as systemic sclerosis, polymyositis/dermatomyositis and mixed connective tissue disease), over-activity of the immune system may result in inflammation and scarring of the lung tissue (this is known as interstitial lung disease which is abbreviated as ILD).

If CTD-ILD is severe and/or progressive, medication to suppress the immune system (immunosuppression) may be required to prevent ongoing lung damage. Currently, our standard of care for severe or progressive CTD-ILD is intravenous (given by drip in to the vein) cyclophosphamide administered monthly for 6 months, followed by oral (tablet) immunosuppression. Occasionally, even this form of intensive immunosuppressive therapy fails to prevent ongoing lung damage and alternative treatments may be required.

Rituximab is another immunosuppressive drug that has proven to be effective in several diseases associated with immune system over-activity (such as rheumatoid arthritis). There is increasing evidence, including experience gained at the Royal Brompton Hospital, to suggest that rituximab may also be effective in treating CTD-ILD when other treatments have failed. The Royal Brompton experience has demonstrated rituximab to be an effective, potentially life-saving medication for the treatment of very severe and progressive CTD-ILD unresponsive to conventional immunosuppression. Intravenous rituximab is administered twice at 2 week intervals.

To determine which drug is best we will compare the results from lung function (breathing) tests and to determine the safety we will compare adverse reactions (side effects). Evaluation of rituximab will involve balancing effectiveness against any side effects.

**Why have I been invited?**

You are being asked to participate in this research study because you have interstitial lung disease associated with a connective tissue disease (systemic sclerosis, polymyositis/dermatomyositis or mixed connective tissue disease) for which your doctor has recommend intravenous cyclophosphamide.

**Do I have to take part?**

No. Taking part in this study is entirely voluntary. If you do decide to take part you will be given this information sheet to keep and you will be asked to sign a consent form. You are free to withdraw from the study at any time without giving a reason. If you decide not to be in the study, or withdraw at any time, this will not affect the clinical care that you receive and you will receive the treatment that your doctor considers the best available for your ILD.

**What will happen to me if I take part?**

If you agree to participate, you will be involved in the study for about 1 year. To make a fair comparison, patients will be randomly allocated to receiving either rituximab or cyclophosphamide. This means neither the doctor nor the patient will know which drug they are receiving. The drugs will be blinded which means the drugs will appear the same and neither you nor your doctor will not know which drug you are receiving. Both drugs are given intravenously and subjects in both groups will be given the same volume at the same visits. To match the two drugs at some visits you will receive a “dummy drug” called a placebo. Patients in the rituximab group will receive a placebo at treatment visits weeks 4/8/12/16/20. Patients in the cyclophosphamide group will receive a placebo at treatment visit day 14. See figure 1. The placebo is a saline (salt water) solution which does not contain any active “drug”.

By participating in the study you will be required to attend the hospital on two occasions when you would not otherwise have to do so were you to simply receive the treatment recommended to you by your doctor. If you participate in the study the visits that you will be asked to make are;

- **Screening Visit(s)**

The first visit will be a screening visit where you will meet the study doctor and nurse who will explain the study and answer your questions. You will be asked to sign a consent form. Several initial tests will be performed including blood tests, urine samples, lung function tests (spirometry) and screening for hepatitis B and C. The results of these tests will be used to ensure that you meet the criteria for participating in the study. If you are taking immunosuppressant drugs (except steroids), you will be required to stop these for 2 weeks before starting the study. This is known as a ‘wash out period’ and if you have to do this, you will need a second screening visit to be assessed for suitability. This wash out period would also be necessary if you were to receive Cyclophosphamide as part of your regular medical care.

Baseline / Treatment 1 visit (Day 0)

At the end of screening your doctor will arrange for you to return to hospital for your first dose of medication. On arrival you will have a number of tests including vital signs (temperature, heart rate, blood pressure and respiratory rate), blood samples, urine samples, lung function test, 6 minute walk test and questionnaires. If you have any signs or symptoms of infection you may be asked to go home and reschedule this visit for when you are well.

Patients who are well enough will be administered the study drug intravenously on the same day. As both types of drugs can cause side effects you will be given a number of drugs to prevent these including MESNA (to prevent bleeding from the bladder), chlorphenamine (an antihistamine to prevent allergy), paracetamol (to prevent fever), hydrocortisone (a steroid to prevent allergy), and ondansetron (an anti-nausea medication). These drugs are given prophylactically to prevent or minimise adverse reactions including nausea, vomiting and allergic reactions to the drugs.

The study drug is given through an intravenous (IV) drip and takes approximately 4-5 hours to administer. After the first dose, we will ask you to remain in on the ward for two hours for observation to ensure you do not have any unexpected side-effects from the drug.

- **Treatment visits 2, 3, 4, 5, 6, 7**

The remaining visits for study drug dosing will also be completed as day visits. These visits will occur at week 2, week 4, week 8, week 12, week 16 and week 20 (see diagram 1). At each visit you will have a number of tests including vital signs (temperature, heart rate, blood pressure and respiratory rate), blood samples, urine samples, lung function test, 6 minute walk test, questionnaires and review by the study nurse and/or doctor to ensure you are well. Each study visit (including tests) will take approximately 4-6 hours.

- **Follow Up Visits 1, 2**

After completing 7 doses of the intravenous study drug, you will undergo assessments to assess the effects of the study drug on your ILD. These will include vital signs (temperature, heart rate, blood pressure and respiratory rate), blood samples, urine samples, lung function test, 6 minute walk test and questionnaires. You will be reviewed by the study nurse and/or doctor to ensure you are well. After the study visit at week 24 it will be up to your doctor to decide on the best treatment for your ILD. This will, in part, depend on your response to treatment and will not be affected by your participation in the trial. In most cases it is likely that you will be recommended to take an oral immunosuppressant medication such as azathioprine. Each of the follow up study visits (including tests) will take approximately 4 hours.

Table 1 summarises what will happen at each study visit.

**Additional Visits**

If you are unwell you may require additional visits to hospital for your doctor to examine you and you may require tests, investigations and possibly treatment. For example, if you have an infection you may require antibiotics. If you are not feeling well enough to receive the study drug, your visit may be rescheduled for when you are better.

**Withdrawal**

If you decide to withdraw from the study prior to receiving all the planned treatment doses, we would still like you to attend the follow up visit at 24 weeks and 48 weeks so that we can measure the effect of any treatment that you have received. If you decide not to undergo any further tests or investigations, we will use the data we have collected up until the point at which you withdraw from the study. You will continue to be followed up routinely by your doctor.

**What will I have to do?**

We ask that you attend the hospital for all the clinic visits. You should tell your study doctor if you have any health problems, even if you think they are not caused by the study medication.

***Before agreeing to take part in this study you should check that any private medical insurance you have will not be affected by your participation.***

You must not take part in any other clinical study while you are participating in this study.

If you have agreed to take part in this study, you should:

- Follow the instructions provided by your study doctor and study staff.
- Tell your study doctor or study staff if you have received another treatment as part of a clinical trial within the previous 8 weeks.
- Tell your study doctor or study staff about any changes in your health or problems that you are having. All participants should be advised to immediately report the onset of any sore throat, bruising, mouth ulcers, nausea, vomiting, abdominal discomfort and dark urine, shortness of breath, or raised temperature to the study team.

Tell your study doctor or study staff about any medications you are taking even if they are obtained without a prescription.

**Study Flow Diagram:**

**Investigations each visit:**

- Blood samples for full blood count, kidney and liver function tests, C-reactive protein, eosinophils sedimentation rate (ESR)
- Mid stream sample of urine for microscopy, culture and sensitivity
- Spirometry
- Observations including temperature, blood pressure, oxygen saturation levels in blood

**Additional investigations at week 0, 12, 24 and 48**

- 6 minute walk test
- Blood samples for immunoglobulin levels and B-cell subsets

**Table 1. Schedule of Events**

|  | **Screening**  **Visit(s)** | **R**  **A**  **N**  **D**  **O**  **M**  **I**  **S**  **E** | **Treatment Visits** | | | | | | | **Follow Up Visit** | |
| --- | --- | --- | --- | --- | --- | --- | --- | --- | --- | --- | --- |
| **visit(s)** | **1 - 2** |  | **1** | **2** | **3** | **4** | **5** | **6** | **7** | **1** | **2** |
| **TIME** | **1-4 wks** |  | **Day**  **0** | **2**  **weeks** | **4 weeks** | **8**  **weeks** | **12**  **weeks** | **16**  **Weeks** | **20**  **weeks** | **24**  **wks** | **48**  **wks** |
| **Consent** | X |  |  |  |  |  |  |  |  |  |  |
| **Study drug** |  |  | X | X | X | x | x | x | x |  |  |
| **Adverse event checking** |  |  | X | X | X | x | x | x | x | x | x |
| **Physical exam** | x |  | x | x | x | x | x | x | x | x | x |
| **Vital signs**  **(pulse, BP, temp, weight)** | x |  | x | x | x | x | x | x | x | x | x |
| **Routine bloods tests** | x |  | x | x | x | x | x | x | x | x | x |
| **Spirometry** | X |  | x | x | x | x | x | x | x | x | x |
| **Blood sample for lymphocyte subsets / Ig level** | X |  |  |  |  |  |  |  |  | x | x |
| **CK (in myositis patients)** | x |  | x | x | x | x | x | x | x | x | x |
| **Ig Levels** | x |  | x |  |  |  | x |  |  | x | x |
| **ECG** | x |  | x |  |  |  | x |  |  | x | x |
| **Lung function tests (DLco)** | x |  | x |  |  |  | x |  |  | x | x |
| **6 MWT*** | x |  | x |  |  |  | x |  |  | x | x |
| **Urinalysis** | x |  | x | x | x | x | x | x | x | x | x |
| **Pregnancy test** | X |  |  |  |  |  |  |  |  |  |  |
| **QoL questionnaires** |  |  | x |  |  |  |  |  |  | x | x |
| **Health economic diary** |  |  | x |  |  |  |  |  |  | x | x |
| **mRSS (scleroderma)** |  |  | x |  |  |  | x |  |  | x | x |
| **Hepatitis B and C serology** | X |  |  |  |  |  |  |  |  |  |  |
| **Blood sample biomarker(s)** |  |  | X |  |  |  | X |  |  | X | X |
| **Blood sample genetics** |  |  | X |  |  |  |  |  |  |  |  |
| **Concomitant medication** | x |  | x | x | x | x | x | x | x | x | x |

Abbreviations; Routine bloods - full blood count, renal and liver function tests, ESR, CRP; Ig levels - immunoglobulin levels, creatinine kinase; ECG – electrocardiogram; HE – Health Economics; mRSS = modified Rodnan Skin Score; 6MWT = 6 minute walk test

Grey shaded square= tests are performed as routine clinical care in patients receiving standard therapy of monthly intravenous cyclophosphamide (for 6 months).

**What are the drugs that are being tested?**

We are comparing the effectiveness of intravenous rituximab with the current standard therapy, cyclophosphamide, as ‘first line’ or initial therapy in patients with CTD-ILD. Intravenous cyclophosphamide has been standard care for severe and/or progressive CTD-ILD for many years. Recent research has shown rituximab may also be a very effective therapy for severe and/or progressive CTD-ILD. This study is to determine which of these two drugs is the most effective. Both drugs are immunosuppressants and work by suppressing the body’s immune system which may make you prone to infections in particular chest and bladder infections.

**What are the alternatives for treatment?**

You do not have to participate in this study to receive treatment for your condition. Other forms of treatment may be available. Your doctor will discuss with you the benefits and risks associated with other choices of treatment. If you do not participate in the study your doctor may still recommend cyclophosphamide for you.

**What medications are not allowed?**

If you are on the study it is recommended you should not be immunized with ‘live’ vaccines such as MMR (measles, mumps and rubella), OPV (oral polio virus), BCG (Bacillus Calmette Guerin – used to prevent tuberculosis) or yellow fever. Discuss any vaccinations with your study team before hand.

We will stop any immunosuppressant drug (except steroids) prior to you being randomised and whilst you are on the study medication.

It is important that you do not take any additional medicines without discussing it first with your study doctor. This includes all other medications even if they are over-the-counter, herbal or homeopathic preparations. If these are required you should only proceed with the permission of the study doctor.

**What are the potential risks and side-effects of the study drugs?**

Like all medicines, cyclophosphamide and rituximab can cause side effects, although not everybody gets them. Most side effects are mild to moderate but some may be serious and require treatment. These drugs act to suppress the immune system in order to prevent further lung damage. A side-effect of suppressing the immune system is increasing the risk of infection (including chest and bladder infections), the majority of which are mild to moderate in severity.

Patients in both treatment groups may receive treatment with the following medications; Mesna, Hydrocortisone, Chlorphenamine, Paracetamol and Ondansetron. These drugs will help to reduce the side effects of nausea, infusion reactions and any discomfort that the patient may encounter during or immediately after the infusions. Normally, patients administered rituximab are given hydrocortisone, chlorphenamine and paracetamol prior to having a rituximab infusion, which all help to prevent or minimise any infusion reactions.

Patients who are prescribed cyclophosphamide in doses > 1000mg are routinely given MESNA to prevent inflammation and or bleeding from the bladder (known as haemorrhagic cystitis). If patients have had prior urethral toxicity the mesna should be given even if the does of cyclophosphamide is equal to or less than 1000mg. Nausea and occasionally vomiting is also a common side effect of cyclophosphamide and patients are routinely given an anti-sickness drug such as Ondansetron for up to 4 days as a preventative against nausea.

Because the drugs being investigated (rituximab and cyclophosphamide) are being blinded, your doctor and nurse will not know which drug you are receiving and therefore all patients will be given Mesna, Hydrocortisone, Chlorphenamine, Paracetamol and Ondansetron.

At each of the study visit you will also be asked about any symptoms and you will have a number of tests and investigations (e.g. blood tests, urine samples, temperature, blood pressure) to check for side effects.

**CYCLOPHOSPHAMIDE**

Common Side Effects reported in more than 1 out of 10 (> 10 %) patients include:

Nausea and occasionally vomiting, mouth ulcers and irregular menstrual cycles in women.

Less Common Side Effects reported in more than 1 out of 100 (approximately 1 - 10%) patients. Cyclophosphamide can cause suppression of the bone marrow, which makes blood cells. Therefore, you could become anaemic (low red blood cells), prone to infection (low white blood cells) or bruise/bleed easily (low platelet count). cyclophosphamide can make you more prone to infection as well as masking the usual symptoms and signs of infection. If you become unwell or develop a temperature while taking cyclophosphamide, you should report to your doctor immediately. If you have not had chickenpox or measles and become exposed to someone with either of them, you should also report to your doctor immediately. Cyclophosphamide can reduce fertility in men and women.

A notable side effect of intermittent high-dose cyclophosphamide treatment is inflammation and bleeding of the bladder (known as haemorrhagic cystitis). It is important that you drink plenty of water (8-10 glasses) on the day and three days after the course of therapy. If you notice any blood in your urine or irritation on passing urine, you should report to your doctor immediately. You will be given a medication (MESNA) to prevent this side effect.

We would like you to be aware that if you get side effects from cyclophosphamide whilst almost all of these are reversible some are not. Ovarian or testicular failure (infertility) has been reported to occur during treatment with cyclophosphamide and in some cases this has been irreversible.

**RITUXIMAB**

Infusion reactions

During or within the first 2 hours of receiving the first infusion you may develop fever, chills and shivering. Less frequently, some patients may experience blisters, itching, sickness, tiredness, headache, breathing difficulties, tongue or throat swelling, itchy or runny nose, vomiting, flushing or palpitations. If you have heart disease or angina, these reactions might get worse.

Tell the person giving you the infusion immediately if you develop any of these symptoms, as the infusion may need to be slowed down or stopped for a while. You will be given drugs prophylactically to prevent or minimise these symptoms. These reactions are less likely to happen after the second infusion.

The most commonly reported in more than 1 out of 10 (> 10 %) patients include: -

Infections such as pneumonia (bacterial), pain on passing water (urinary tract infection), allergic reactions after infusion, changes in blood pressure, nausea, rash, fever, feeling itchy, runny or blocked nose and sneezing, shaking, rapid heart beat, tiredness and headache

Less common side effects reported in more than 1 out of 100 (1 – 10 %) patients include:-

Infections such as bronchial tube inflammation (bronchitis), a feeling of fullness or a throbbing pain behind the nose, cheeks and eyes (sinusitis), pain in the abdomen, vomiting and diarrhoea, breathing problems, high cholesterol levels in the blood, abnormal sensations of the skin such as numbness, tingling, pricking or burning, sciatica, migraine, dizziness. Also anxiety, depression, indigestion, diarrhoea, acid reflux, irritation and /or ulceration of the throat and the mouth, pain in the tummy, back, muscles and/or joints.

Uncommon side effects reported in more than 1 out of 1000 (0.1 – 1 %) patients include:

Excess fluid retention in the face and body, inflammation, irritation and / or tightness of the lungs and throat, coughing, skin reactions including hives, itching and rash, allergic reactions including wheezing or shortness of breath, swelling of the face and tongue, collapse.

Very rare side effects include a reduction in immunoglobulins (proteins that help fight infection) in the blood and a particular neurological disorder called progressive multifocal leukoencephalopathy (PML). If this develops individuals may notice neurological symptoms (e.g. weakness, numbness, and tremor) or poor concentration, forgetfulness or confusion. If you become aware of such symptoms you must alert the study doctor immediately.

MESNA

Because patients receive potent cytotoxic drugs concurrently, the side-effect profile of MESNA is difficult to define. In healthy volunteers the following side effects occurred following single doses: nausea, vomiting, colic, diarrhoea, headache, fatigue, limb and joint pains, depression, irritability, lack of energy, rash, hypotension and tachycardia. In rare cases allergic reactions have been reported.

Hydrocortisone

Given at prolonged high doses, steroids can have a number of significant side effects. Given as a single low dose intravenously prior to rituximab the chances of adverse effects arising due to hydrocortisone is low. Possible side effects include; transient mood disturbances, sleep disturbance (usually only on the night following injection), allergic reactions (symptoms include itching or skin rashes, swelling of the face, lips or throat, difficulty in breathing or wheeziness), elevated blood sugar and a short term rise in blood pressure.

Chlorphenamine

Chlorphenamine is available over the counter in pharmacies and is most commonly used as a treatment for hay fever and minor allergies. Although very safe, it has been reported to cause occasional side effects. These include; allergic reactions**,** sedation (with a single dose this effect is usually minor), nausea and diarrhoea.

Paracetamol

Paracetamol is a commonly used pain killer that is widely available for purchase. Used at prescribed doses it is an incredibly safe drug. Very occasional side effects have been reported and these include rashes, bruising and wheeze.

Ondansetron

Ondansetron is an anti-sickness medication. It is commonly used and is considered to be very safe. The most commonly reported (in about 1 in 10 people) is headache. Other relatively common side effects (affecting about 1 in 100 people) are constipation and a sense of flushing.

**Are there any benefits to me if I participate in the study?**

If you participate in the study there is no guarantee of any benefit to you. The information gathered from the study will provide important information that may be used to improve the treatment of future patients with CTD-ILD. Treatment of your ILD may also have benefits for other symptoms of your connective tissue disease, such as reduced joint pain, reduced muscle weakness and improved skin thickness.

You will not be paid for taking part in this study although reasonable travel expenses will be paid where necessary for visits that occur in excess to your normal clinical care (that is any screening visits and the day 14 visit).

You will not be asked to pay for any treatment costs associated with the study protocol or follow-up visits.

**What are the possible disadvantages and risks of taking part?**

As in any research study the use of the study treatments may be associated with certain unforeseen risks. It is very important that you report any side effect you may experience to your study doctor. If you become unwell or are injured as a direct result of the trial drug or any aspect of the trial procedures performed during your participation in this research trial, please contact your study doctor.

In addition to the risks associated with the study drugs you may experience some discomfort as a result of the other tests and investigations you will have.

**Blood samples and intravenous injections:** Pain, tenderness and bruising, and on rare occasions infection at the puncture site and some individuals may feel light headed at the time. Some people may be sensitive to the tapes used to secure the IV injection.

**6 minute walk test:** No serious risk associated with this test

**Lung function tests:** No serious risk associated with this test

**ECG:** No serious risk associated with this test, occasionally a skin reaction to the electrodes.

**Pregnancy or Breast Feeding**

Both men and women should avoid starting a family during and for 12 months after last study medicine infusion. Pregnant women or women who plan to become pregnant must not take part in this study as the study medications have not been approved for use in pregnancy and may therefore cause harm to an unborn child and result in birth defects. If you are a woman or partner of a woman of child bearing potential, you must be using a medically approved method of birth control for the duration of the study and for a period of at least 12 months after last study medicine infusion. You should use either a barrier method, i.e. condom or occlusive cap with spermicide or vasectomized partner, or a highly effective non-barrier method including oral, injected or implanted hormonal contraceptives and intrauterine device or system. Please consult with your doctor if you have any questions.

Female patients will be considered of childbearing potential unless surgically sterilized by hysterectomy or bilateral tubal ligation, or if they have been post-menopausal for at least two years. With surgical sterilization or tubal ligation, your partner must use a condom, unless you are post-menopausal. If you believe yourself to have been post-menopausal for at least two years but you are a bit uncertain, use contraception as described.

All female patients of childbearing potential must undergo a pregnancy test prior to commencing the first treatment. A pregnancy test can only reliably confirm pregnancy some days after conception. **Please inform your doctor immediately, should you become pregnant or suspect that you may be pregnant.** You cannot take part in the study if you are breastfeeding an infant.

Should you become or think that you have become pregnant during the study, **you must immediately tell your study doctor.** Your study treatment will be stopped immediately and your further treatment will be discussed. Your health and your baby’s health will be monitored throughout your pregnancy and the outcome will be reported to the study sponsor.

**Ionising Radiation (Medical Exposure) regulations- IRMER**

No additional x-rays, CT scans or exposure to radiation will occur as a result of taking part in this study. Patients will have had a CT scan within 12 months of being included in the study but this is part of routine clinical evaluation.

**What happens when the research study stops?**

At the end of the research your care will continue as usual with the medical team at your hospital. You are likely to be commenced on azathioprine or another oral immunosuppressant medications when the study drug regime is stopped at the post study follow-up visits. You will continue on this until instructed otherwise by your medical team. Your medical team will decide on your future treatment. After the end of study follow-up visit we will not ask you to attend for any more visits or any more procedures as part of the study. You will be asked to attend your usual out patient appointments.

**Part 2**

**What if relevant new information becomes available?**

Sometimes during the course of a research study, new information becomes available about the treatment that is being studied. If this happens, we will tell you about it and discuss with you whether you want to continue in the study. If you decide to withdraw we will make arrangements for your care to continue. If you decide to continue in the study you will be asked to sign an updated consent form. Also, on receiving new information we might consider it to be in your best interests to withdraw you from the study. We will explain the reasons and arrange for your care to continue.

**What if I do not want to carry on with the study?**

Your participation in this study is voluntary and you may withdraw from the study at any time without prejudice to your future medical care. Should you decide to withdraw from the study for any reason, you are asked to contact the study team immediately. Should your participation in the study be terminated, regardless of the reason, you will not suffer any penalties or loss of benefits to which you are otherwise entitled.

If for any reason you decide to withdraw from the study earlier than planned, the study sponsor will wish to keep the data that has already been collected and the study team may ask for your permission to contact you, your GP or hospital medical team periodically to gather general information about your health after stopping study medication. Although you are under no obligation to give the reasons for stopping study medications, it may help the study team or benefit others if this information is made available.

**Will my taking part in the study be kept confidential?**

If you join the study, your medical records and the data collected for the study may be reviewed by authorised persons from the Royal Brompton & Harefield NHS Foundation Trust who are the Sponsor of the study and are organising and managing the study. They may also be reviewed by authorised persons from the UK Medicines Healthcare Regulatory Authority or other NHS organisations where it is relevant to your taking part in research to check that the study is being carried out correctly. All personnel will have a duty of confidentiality to you as a research participant. Any information about you which leaves the hospital will have your name, address and personal details removed so that you cannot be recognised from it and will be identifiable to only the study team.

**Will my General Practitioner/Family doctor (GP) be informed of my involvement?**

With your consent your GP will be informed that you are participating in the study and kept informed of your medical progress. We may exchange information regarding your general medical health with your GP.

**What will happen to the results of the research study?**

At the end of the project all the research results are gathered together and analysed. The researchers have a professional responsibility to publish their findings, however your identity will not be revealed. Most research is published in the medical press and if you are interested in knowing the overall results of the study, ask the researchers about this. You are entitled to see any results or information held about you under the Freedom of Information Act.

***Participation in the RECITAL Biomarker and Genetic research***

If you agree to participate in the study you will also be invited to participate in the biomarker and genetic part of the study. By looking at DNA (genes) and proteins (biomarkers) in your blood, researchers may be better able to understand how patients differ in the way they respond to Cyclophosphamide or Rituximab. Both DNA and biomarkers are obtained from a simple blood test. We are trying to find new biomarkers and identify genes that may help us treat CTD-ILD disease better. Approximately six samples of 10mls (about 2 teaspoonfuls) of blood will be taken by a trained person during the study. Your sample will be given a code, so only the study site can link it back to you, and will be kept in locked storage. Samples will be collected and analysed at either the Royal Brompton Hospital (RBH), Imperial College London, a commercial research organization (CRO) or in collaborating academic institutions. Analysis of these samples may be undertaken after completion of the study and following assessment of the primary study outcome.

Research blood sample data will be held on a secure database under the custodianship of the Chief Investigator.

Participation in the RECITAL Biomarker and Genetic research is voluntary. If you decide to take part, you are still free to withdraw at any time if you change your mind. The sponsor may store your samples in a Biobank (a repository of samples). Alternatively, the sponsor may destroy your sample in accordance with the Human Tissue Act before then. If you decide not to take part or to withdraw your consent after starting the Biomarker and/or Genetic part of the study, you do not have to give a reason and there will be no change to your medical treatment or to your participation in this study.

If you withdraw from the Biomarker and/or Genetic research, your sample will be destroyed and the sponsor will only keep any study information collected/generated up to that point. If you withdraw from the clinical study, you will be given the option to retain your Biomarker and/or Genetic sample or to have your Biomarker and/or Genetic sample(s) destroyed.

In the unlikely case that there is a problem processing your sample, then we may ask you to give a second sample. In special cases, your sample may not be used. This might happen if there are not enough subjects, if the study is stopped for other reasons, or if no questions are raised about how people respond to cyclophosphamide or rituximab.

We would like to use your sample for scientific and medical research purposes for example, to test for certain proteins and other biological markers of disease and prognosis. Please be aware that your consent allows extensive use of your coded data. We ask you to explicitly confirm your consent for the testing of your blood samples by putting your initials where appropriate on the consent form.

The purpose of these analyses is not to provide a diagnosis for you. Results from the genetic analyses will not be used to prove any disease-causing genes which may carry any risk of disease for you or your close relatives. The intention is rather to determine genetic associations related to connective tissue disease, ILD or response to treatment.

***What benefits can I expect from the Biomarker and Genetic part of the study?***

If you take part in the Biomarker and/or Genetic part of the study there is no direct benefit to your self. However you may help researchers understand why people react differently to cyclophosphamide or rituximab.

**Who is organising and funding the research?**

The study is being organised by the Royal Brompton and Harefield NHS Foundation Trust. The study is funded by the Medical Research Council through its efficacy and mechanism evaluation program. The doctors conducting the research are not being paid for including you in the study. However, your hospital will receive some reimbursement to cover the cost of the additional work required to carry out this study.

**Who has reviewed the study?**

The study has been reviewed by a team of experts to ensure that it meets the highest standards of quality and care. This study has been given a favourable ethical opinion by London Westminster ethics committee.

**Who can I contact if I have further questions or if a problem occurs?**

If you would like any further information about the study, either now or at time during the course of the study, please ask a member of the Research Team.

Principal Investigator

Name

Telephone

Study Nurse/coordinator

Name

Telephone

**What if there is a problem?**

If you have a concern about any aspect of the study, you should ask to speak with the researchers who will do their best to answer your questions and resolve any queries. Their contact details are provided in this patient information sheet.

If you remain unhappy and wish to complain formally you can do this through the National Health Service Complaints Procedure. The Patient Advice and Liaison Service (PALS) can help you with this:

**PALS Name:**

**Tel:**

**Email:**

**IF YOUR DOCTOR IS NOT ACCESSIBLE FOR ANY REASON, AN ALTERNATE SHOULD BE CONTACTED IMMEDIATELY.**

Dr Toby Maher

Consultant Physician

Royal Brompton Hospital

Sydney Street

London SW3 6NP

Phone: 020 7352 8121

Secretary: 020 7351 8018

Fax: 020 7351 8951

**Thank you for taking the time to consider this study. If you do choose to participate, you will be given a copy of this information sheet to keep and also a copy of the consent form that you will be asked to sign.**
