# Supplementary material for: Rituximab versus cyclophosphamide for the treatment of connective tissue disease-associated interstitial lung disease (RECITAL): study protocol for a randomised controlled trial
Source: Trials. 2017 Jun 15;18:275. doi: 10.1186/s13063-017-2016-2 (PMC5471887; doi:10.1186/s13063-017-2016-2)
Supplement: Supplementary file 4 — Informed Consent Form. Version 6.0. 15.10.2014. (DOC 39 kb) [file 13063_2017_2016_MOESM4_ESM.doc]

*To be printed on Site headed paper*

Centre Number:

Study Number:

Subject ID:

**INFORMED CONSENT FORM**

**Title of Project:** A randomized, double blind controlled trial comparing Rituximab against intravenous Cyclophosphamide in connective tissue disease associated interstitial lung disease (RECITAL Study)

**Name of Principal Investigator:** <Name>

**Please INITIAL box if you agree**

| 1 | I confirm that I have read and understand the information sheet dated 15^th^ October 2014 (Version 6.0) for the above study and that the study has been explained to me in a language that I understand. | ⬜ |
| --- | --- | --- |
| 2 | I have had the opportunity to consider the information, ask questions and have had these answered satisfactorily. | ⬜ |
| 3 | I have been given names and contact details of study staff whom I can call. | ⬜ |
| 4 | I understand that my participation is voluntary and that I am free to withdraw at any time, without giving any reason and without my medical care or legal rights being affected. | ⬜ |
| 5 | I understand that where it is relevant to my taking part in this research, relevant sections of my medical notes and data collected during the study, may be looked at by responsible individuals from the Sponsor the Royal Brompton & Harefield NHS Foundation Trust, from regulatory authorities or from the NHS Trust where this study is being conducted. I give permission for these individuals to have access to my records. Should I withdraw from the study, I understand that the data collected up to that point will still be processed and analysed. | ⬜ |
| 6 | I agree to my GP, and when appropriate, my local consultant being informed of my participation in the study. | ⬜ |
| 7 | I agree to my data being kept for at least 5 years after termination or discontinuation of the trial, as specified in the regulation relating to clinical trials with medicinal products. After that period I understand that my personal data will be deleted assuming that there are no opposing legal, constitutional or contractual retention periods. | ⬜ |
| 8 | I understand that my medical data, where it is relevant to this study, will be used for scientific research and that this will be published. I agree to this, under the condition that my privacy will be adhered to, and that the study results will be processed anonymously. I give permission to record my participation in this study in my medical notes. | ⬜ |
| 9 | I understand that samples that I donate, including DNA samples, will be analyzed, anonymously, by researchers outside my local hospital, potentially including overseas. I also understand that after the end of the study samples will be stored and may be used, anonymously, in future ethically approved research. | ⬜ |
| 10 | I know that I cannot be in another study while I am taking part  in this study | ⬜ |
| 11 | I freely agree to take part in the above research | ⬜ |

**Name of Investigator / Researcher**

Signed Dated

Print Name

**Patient consent**

Signed Dated

Print Name

**Thank you for agreeing to take part in this study**

**A copy of the signed consent must be given, along with the Participant Information Sheet (PIS), to the participant. A copy must be filed in the participant’s medical records and a copy must be retained in the Investigator Site File (ISF).**
