# Supplementary material for: Rituximab versus cyclophosphamide for the treatment of connective tissue disease-associated interstitial lung disease (RECITAL): study protocol for a randomised controlled trial
Source: Trials. 2017 Jun 15;18:275. doi: 10.1186/s13063-017-2016-2 (PMC5471887; doi:10.1186/s13063-017-2016-2)
Supplement: Supplementary file 5 — Exclusion criteria. (DOCX 90 kb) [file 13063_2017_2016_MOESM5_ESM.docx]

# Exclusion Criteria

- Age <18 or >80 years.
- Previous treatment with Rituximab and/or intravenous Cyclophosphamide
- Known hypersensitivity to Rituximab or Cyclophosphamide or their components
- Significant (in the opinion of the investigator) other organ co-morbidity including cardiac, hepatic or renal impairment
- Co-existent obstructive pulmonary disease (*e.g.* asthma, COPD, emphysema) with pre bronchodilator FEV1/FVC < 70%
- Patients at significant risk for infectious complications following immunosuppression
  - Including HIV positive or other immunodeficiency syndromes (including hypogammaglobulineamia)
- Suspected or proven untreated tuberculosis
- Viral hepatitis
- Infection requiring antibiotic treatment in the preceding four weeks
- Unexplained neurological symptoms (which may be suggestive of progressive multifocal leukoencephalopathy;PML). Neurological symptoms arising as a consequence of the underlying CTD do not necessitate exclusion.
- Other investigational therapy (participation in research trial) received within 8 weeks of randomisation
- Immunosuppressive or CTD disease modifying therapy (other than corticosteroids) received within 2 weeks of randomisation
- Pregnant or breast feeding women, or women of child-bearing potential, not using a reliable contraceptive method for up to 12 months following IMP
- Unexplained haematuria, or previous bladder carcinoma
- CT scan > 12 months from randomisation
